# Synthesis of 1,2-*cis*-2-*C*-branched aryl-*C*-glucosides via desulfurization of carbohydrate based hemithioacetals

**DOI:** 10.3762/bjoc.11.64

**Published:** 2015-04-29

**Authors:** Henok H Kinfe, Fanuel M Mebrahtu, Mandlenkosi M Manana, Kagiso Madumo, Mokela S Sokamisa

**Affiliations:** 1 Department of Chemistry, University of Johannesburg, PO Box 524, Auckland Park 2006, South Africa

**Keywords:** aryl-*C*-glucoside, desulfurization, Ferrier product, hemithioacetal

## Abstract

1-*C* and 2-*C*-branched carbohydrates are present as substructures in a number of biologically important compounds. Although the synthesis of such carbohydrate derivatives is extensively studied, the synthesis of 1,2-*cis*-2-*C*-branched *C*-, *S*-, and *N*-glycosides is less explored. In this article a synthetic strategy for the synthesis of 1,2-*cis*-2-*C*-branched-aryl-*C*-glucosides is reported via a hydrogenolytic desulfurization of suitably orientated carbohydrate based hemithioacetals. 1,2-*cis*-2-Hydroxymethyl and 2-carbaldehyde of aryl-*C*-glucosides have been synthesized using the current strategy in very good yields. The 2-carbaldehyde-aryl-*C*-glucosides have been identified as suitable substrates for the stereospecific preparation of 2,3-unsaturated-aryl-*C*-glycosides (Ferrier products).

## Introduction

1-*C* (C-glycosides) and 2-*C*-branched carbohydrates are important carbohydrate analogues which have found wide application in glycochemistry and medicinal chemistry [1–5]. *C*-Glycosides, especially aryl-*C*-glycosides, are the main structural features of a number of biologically active natural products such as pluramycins (antibacterial and antitumor activities), angucyclines (antibacterial, antitumor activities, and inhibitors of oxidative enzymes), and benzoisochromanequinones (antibacterial, antitumor and antiplatelet aggregation activities) [2]. Moreover, the stability of the C–C glycosidic bond in *C*-glycosides provides the potential to serve as inhibitors of carbohydrate degrading enzymes [1–5]. Owing to their profound biological importance, there are different routes reported for their synthesis but the common ones are: nucleophilic substitution of an activated glycosyl donor with organometallic aryl derivatives, cross-coupling of aryls with glycals, Friedel–Crafts arylation at the anomeric centre of an activated glycosyl donor, and rearrangement of aryl-*O*-glycosides to their corresponding aryl-*C*-glycosides [1–6]. Most of these methods provide 1,2-*trans* aryl *C*-glycosides and formation of 1,2-*cis*-aryl-*C*-glycosides, especially α-glucosides, still remains a challenge.

Equally important to *C*-glycosides are 2-*C*-branched carbohydrates which are employed as bioisosters of the natural 2-acetamido glycoside derivatives. Gammon et al. prepared a series of 2-*C*-branched carbohydrates as potential inhibitors of enzymes implicated in the biosynthesis of mycothiol, *Mycobacterium tuberculosis’s* defensive low molecular weight thiol [7]. 2-*C*-acetamide and 2-*C*-acetonyl carbohydrate derivatives have also been reported to serve as inhibitors of the biosynthesis of lipids [8–9] and cell surface engineering [10], respectively. The main synthetic protocols for the synthesis of 2-*C*-branched sugars involve the regioselective ring opening of 1,2-cyclopropanated carbohydrates and the radical addition reaction of glycals [11–15].

Although the synthetic methodologies developed for the synthesis of *C*-glycosides and 2-*C*-branched sugars are extensively studied, the synthesis of 1,2-*cis*-2-*C*-branched *C*-, *S*-, and *N*-glycosides is less explored [11]. Herein, we wish to report a stereoselective strategy for the synthesis of 1,2-*cis*-2-*C*-branched aryl-*C*-glucosides.

## Results and Discussion

**Scheme 1:**
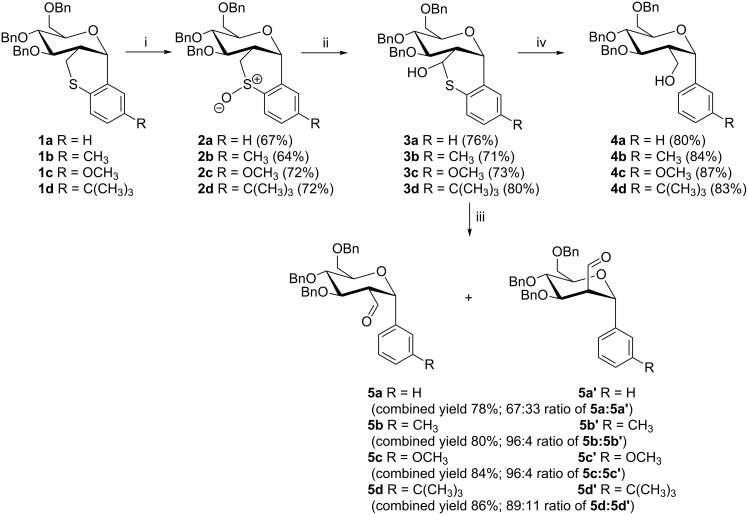
(i) CAN, wet silica, KBr, CH_2_Cl_2_/CH_3_CN (1:1), rt, 30 min; (ii) a. NaOAc, Ac_2_O, 140 °C, 3 h; b. K_2_CO_3_, CH_3_OH, rt, 10 min; (iii) W-1 Raney nickel, acetone, rt, 45 min; (iv) NiCl_2_·6H_2_O, NaBH_4_, MeOH/THF (11:4), 0 °C, 10 min.

**Figure 1:**
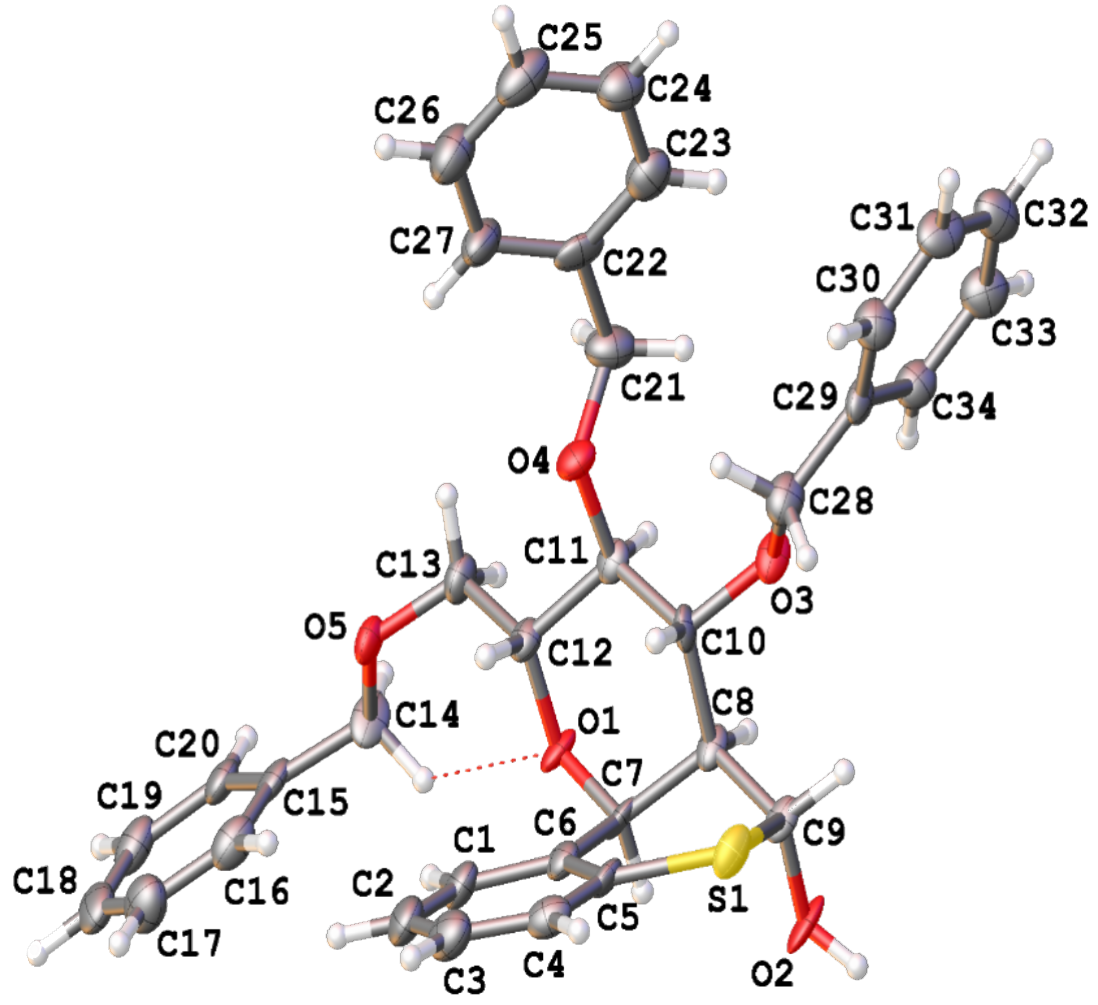
Single X-ray crystal structure of hemithioacetal **3a**.

We recently reported on the stereoselective synthesis of carbohydrate based thiochroman **1** whereby the C-1 and C-2 substituents of the sugar moiety are locked in a 1,2-*cis* configuration in the thiochroman ring [16–17]. With our long standing on the chemical transformation of the thiochromans into important intermediates and biologically active compounds [16–19], it was noted that hemithioacetal **3** can be readily prepared from thiochroman **1** and we envisioned that a careful desulfurization could provide aryl-*C*-glucoside **4** with inherent 1,2-*cis* relationship between the easily transformable 2-*C*-branch and the aryl group at the C-2 and C-1 positions, respectively. Although detailed studies on the desulfurization of hemithioketals are well documented, the desulfurization of the related hemithioacetals (RCH(OH)SR) are less explored [20–21]. In achieving our goals, sulfoxide **2a** synthesized from a controlled oxidation of thiochroman **1a** was subjected to Pummerer rearrangement followed by Zemplén deacetylation to provide hemithioacetal **3a** (Scheme 1). During the deacetylation reaction the hemithioacetal **3a** precipitated out of the methanol solution. Although most hemithioacetals (RCH(OH)SR’) are usually unstable and difficult to isolate as they dissociate to their corresponding aldehydes and thiols [22], hemithioacetal **3a** was found to be stable and its structure was confirmed by X-ray crystal diffraction analysis (Figure 1) [23].

After the successful synthesis and characterization of the hemithioacetal **3a**, our initial desulfurization attempt to prepare the 2-*C*-branched-α-aryl-*C*-glucoside **4a** involved treatment of a solution of hemithioacetal **3a** in ethanol with freshly prepared Raney Nickel (W-2). However, the reaction led to the formation of the desired product in un-usable quantities along with randomly de-benzylated and other byproducts. Attempts for the exclusive synthesis of glucoside **4a** using different strengths of Raney nickel and reaction conditions (solvent, temperature and reaction time) were all unsuccessful. Interestingly, during the course of the investigation it was noted that the reaction proceeded via the formation of a less polar intermediate (judged by TLC analysis). This intermediate was properly isolated and detailed NMR and HRMS studies indicated that the intermediate was an inseparable mixture of carbaldehydes **5a** and **5a’**. Evidence for carbaldehyde **5a** include the appearance of the aldehydic proton at δ 9.58 as a doublet (*J* = 2.4 Hz) due to coupling to H-2. The anomeric proton appeared as a doublet with a coupling constant *J* of 2.8 Hz which corresponds to an axial–equatorial relationship between H-1 and H-2 suggesting an α-configuration of the glycosidic bond [24–25]. Similarly, the aldehydic and anomeric protons of carbaldehyde **5a’** appeared as doublets at 9.79 and 5.21 ppm with 2.0 and 5.2 Hz coupling constants, respectively. The assignment of the glucoside **5a** and mannoside **5a’** was based on a prolonged treatment of the aldehydic mixture with trifluoroacetic acid which effected no change in the ratio of the two epimers suggesting that it represents an equilibrium mixture. Consequently, this led us to the conclusion that the major isomer is carbaldehyde **5a** which contains the minimum number of axial substituents (more stable).

**Scheme 2:**
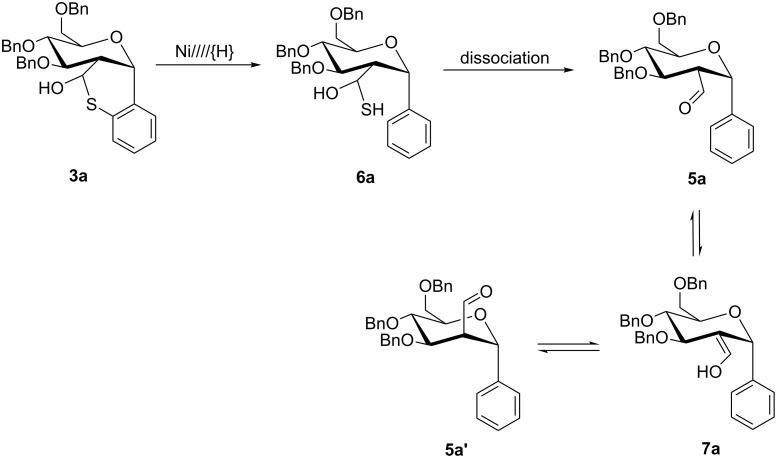
Proposed reaction sequence for the synthesis of a mixture of carbaldehydes **5a** and **5a’** using Raney nickel, Ni////{H}, as a desulfurizing agent.

Upon further investigation we found that carbaldehydes **5a** and **5a’** (3:1 ratio) could be exclusively prepared (as mixture) by treatment of hemithioacetal **3a** with W-1 Raney Nickel in acetone at ambient temperature with no evidence on the formation of glucoside **4a** and ring contraction by sulfur extrusion. Consideration of the conditions required for the formation of the mixture of carbaldehydes **5a** and **5a’** suggests that hydrogenolytic desulfurization of the C(sp^2^)-S bond of hemithioacetal **3a** followed by dissociation/decomposition of the resulting thioaldehyde hydrate **6a** yields carbaldehyde **5a**. Keto–enol tautomerization (equilibration) of carbaldehyde **5a** via enol **7a** might have then resulted in the formation of carbaldehyde **5a’**, as shown in Scheme 2.

In order to investigate the generality of the protocol, several hemithioacetals (**3a–d**) were synthesized and subjected to the desulfurization reaction (Scheme 1). The hemithioacetals were transformed into their corresponding carbaldehydes **5** in excellent yields. It is noted that in the presence of substituents at the aromatic group of the C-1, the selectivity towards the formation of 2-carbaldehyde glucoside isomers is favoured due to the increased steric hindrance for the equatorial approach and the presence of fewer axial substituents.

**Scheme 3:**
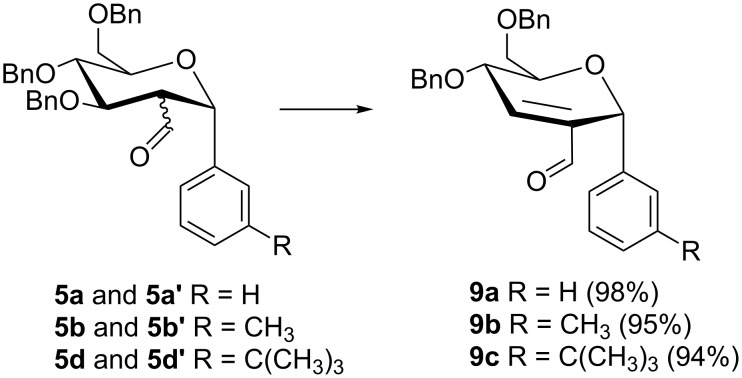
K_2_CO_3_ (catalytic amount), MeOH, rt, 30 min.

To explore their synthetic potential, the mixture of carbaldehydes **5a** and **5a’** was treated with a catalytic amount of K_2_CO_3_ according to Scheme 3 to afford 2,3-unsaturated-α-aryl-*C*-glycoside **9a** (Ferrier product) which was claimed to have been synthesized under acidic conditions [26] by a completely different reaction protocol. However, our spectroscopic data were not in agreement with the reported data [26]. Firstly, the integration of the protons reported in the literature does not correspond to the number of hydrogen atoms present in the Ferrier product **9a**; secondly, the aldehydic carbonyl carbon appeared at 200.3 ppm which is off downfield by 10 ppm relative to the carbonyl carbons of a similar series of analogues reported in the same communication and a related report by Gammon et al. [27] (this carbon appeared at around 191 ppm in all of the other reported Ferrier products); thirdly, H-1 is reported to appear as a doublet with a coupling constant *J* of 4.8 Hz which is too large for a possible long range coupling with H-3. On the contrary, the aldehydic carbon appeared at 191 ppm while H-1 resonated at 5.55 ppm as a singlet in the current report. The appearance of the aldehydic proton and the H-1 as singlets, H-3 downfield in the aromatic region as well as the integration of the aromatic protons and benzylic protons to 15 and 4, respectively, indicate the abstraction of the H-2 and elimination of the benzyloxy group at C-3. The absence of a coupling between H-1 and H-5 in the NOE NMR spectrum confirmed the α-configuration of the glycosidic bond at C-1. The formation of a single Ferrier product from a mixture of starting materials indirectly confirmed that carbaldehydes **5a** and **5a’** were indeed epimers at C-2.

In order to evaluate the generality and scope of the proposed synthesis of the 2,3-unsaturated-α-aryl-*C*-glycosides, the reaction was monitored with hemithioacetals **5** (Scheme 3). The reaction provided the corresponding 2,3-unsaturated-α-aryl-*C*-glycosides **9a–c** in excellent yields (Scheme 3). The high stereospecificity for an α-anomer, mild, non-acidic (alkaline) reaction conditions as well as absence of the need for transition metal reagents makes the current protocol a viable alternative to the literature reported methodologies.

After the serendipitous and successful exclusive synthesis of carbaldehydes **5** using W-1 Raney nickel, we wondered if we could tailor the reaction so that the glucoside **4** would be formed stereospecifically with the use of other desulfurizing agents but starting with the same hemithioacetal **3** substrate as it was originally proposed. To our delight, treatment of a solution of hemithioacetal **3a** and nickel chloride hexahydrate in a mixture of methanol and tetrahydrofuran with sodium borohydride at 0 °C following the protocol reported by Back et al. [28–29] provided the desired glucoside **4a** in 80% yield (Scheme 1). Hemithioacetals **3a–d** were transformed into their corresponding glucosides **4a–d** in moderate to excellent yields (Scheme 1). In all cases, no other significant product was identified upon TLC analysis and in the NMR spectra of the crude products. However, no plausible mechanism as in the case of the synthesis of carbaldehydes **5** could be postulated since the reaction proceeded too fast to allow for the identification and isolation of possible intermediates to give insight into a possible mechanism.

## Conclusion

In conclusion, we have demonstrated that desulfurization of carbohydrate based hemithioacetals **3** allows for the stereoselective synthesis of 2-*C*-branched 1,2-*cis*-aryl-*C*-glucosides. The method has been applied to the synthesis of either 1,2-*cis*-2-hydroxymethyl or 2-carbaldehyde of aryl-*C*-glucosides from a common starting material. Compared to the previously reported protocols, especially via opening of 1,2-cyclopropanated sugars [30–31], the current strategy is superior in stereoselectivity and amenability of the 2-*C*-branch for further manipulation. The synthetic application of the 2-carbaldehyde of the aryl-*C*-glucosides was demonstrated by their ease of stereospecific transformation into the 2,3-unsaturated-α-aryl-*C*-glycosides (Ferrier products) without the need for acid catalysts or transition metal-based reagents as normally required. The unexpected exclusive formation of carbaldehydes **5** is expected to shed light into the understanding of the mechanism of desulfurization using Raney nickel. The use of these 2-*C*-branched 1,2-*cis*-aryl-*C*-glucosides, having the required orientation at the anomeric and C-2 positions, in the synthesis of compounds that will mimic biologically active compounds that are substrates for the enzymes of *Mycobacterium tuberculosis*, in particular a deacetylase implicated in the biosynthesis of mycothiol, is under way and the results will be reported in the future.

## Supporting Information

File 1 Full experimental details.

File 2 ^1^H and ^13^C NMR spectra.
