# Supplementary material for: Synthesis of 1,2-cis-2-C-branched aryl-C-glucosides via desulfurization of carbohydrate based hemithioacetals
Source: Beilstein J Org Chem. 2015 Apr 29;11:583–8. doi: 10.3762/bjoc.11.64 (PMC4464409; doi:10.3762/bjoc.11.64)
Supplement: File 1 — Full experimental details. [file Beilstein_J_Org_Chem-11-583-s001.pdf]

Supporting Information

for

Synthesis of 1,2-*cis*-2-*C*-branched aryl-*C*-glucosides via desulfurization of carbohydrate based hemithioacetals

Henok H. Kinfe*, Fanuel M. Mebrahtu, Mandlenkosi M. Manana, Kagiso Madumo and Mokela S. Sokamisa

Address: Department of Chemistry, University of Johannesburg, PO Box 524, Auckland Park 2006, South Africa

*Corresponding author

Email: Henok H Kinfe - hhkinfe@uj.ac.za

Full experimental details

General methods

All solvents were dried by appropriate techniques reported in the Purification of Laboratory Chemicals by Perrin and Armarego [1]. Thiochromans **1** and sulfoxides **2** were synthesized according to previously published protocols [2,3]. All reactions were monitored by thin-layer chromatography (TLC) on aluminum-backed silica gel 60 F254 plates using an ascending technique. The plates were visualized by spraying with a 1:1 solution of 5% *p*-anisaldehyde in ethanol and 10% sulfuric acid in ethanol then baking at 150 °C. Gravity column chromatography was done on silica gel 60 (70–230 mesh). Optical rotations were determined in chloroform solutions at 25 °C. The concentration *c* refers to g/100 mL. All ^{1}H and $^{13}C\{^{1}H\}$ NMR spectra were recorded as deuteriochloroform solutions using tetramethylsilane as an internal standard. All chemical shifts are reported in ppm.

General procedure for the synthesis of hemithioacetals 3a–c

A stirred mixture of sulfoxide **2** (0.29 mmol) and sodium acetate (0. 05 mmol) in acetic anhydride (1 mL) was refluxed at 140 °C for 3 h. The reaction was allowed to cool to room temperature. Diethyl ether (5 mL) and methanol (1 mL) were added to the reaction mixture and stirred for 1 h. The solution was concentrated at reduced pressure. The residue was diluted with dichloromethane and washed several times with saturated aqueous sodium bicarbonate solution. The organic layer was dried over $MgSO_4$, concentrated in vacuo to give a yellow oil. The product was pure enough to be carried to the next step without further purification. To a solution of the yellow oil (0.285 mmol) in methanol (5 mL) potassium carbonate (0.029 mmol) was added and stirred vigorously at room temperature for 10 min. The white precipitate formed was filtered and washed several times with water. Further crops of the title product were collected by extraction of the aqueous filtrate with ethyl acetate (3 × 10 mL). The combined organic phases were dried over $MgSO_4$, concentrated in vacuo and the

crude product was purified by column chromatography using a mixture of ethyl acetate:hexane (2:8) as an eluent to provide hemithioacetals **3a–d:**

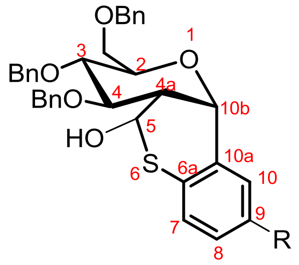

(2*R*,3*S*,4*R*,4a*S*,5*S*,10b*S*)-3,4-bis(benzyloxy)-2-[(benzyloxy)methyl]-2,3,4,4a,5,10b-hexahydrothiochromeno[4,3-*b*]pyran-5-ol (3a): 76% yield; white crystals; mp 128-131 °C; $[\alpha]_D$ (*c* 0.1, $CHCl_3$) +5.0; IR (neat cm^{-1}) 3320, 3030, 2911, 1497, 1355, 696; ^1H NMR (400 MHz, $CDCl_3$): δ 7.67-7.62 (m, 1H, Ar), 7.42-7.25 (m, 13H, Ar), 7.16-7.04 (m, 5H, Ar), 5.47 (d, *J* = 4.0 Hz, 1H, H-5), 5.36 (d, *J* = 6.0 Hz, 1H, H-10b), 4.95 (d, *J* = 11.2 Hz, 1H, CH_AH_BPh), 4.81-4.71 (m, 3H, CH_AH_BPh, 2 x CH_AH_BPh), 4.59 (d, *J* = 12.4 Hz, 1H, CH_AH_BPh), 4.54 (d, *J* = 10.8 Hz, CH_AH_BPh), 3.84-3.68 (m, 4H, H-1`a, H-1`b, H-3 and H-4), 3.59-3.51 (m, 1H, H-2), 2.78-2.69 (m, 1H, H-4a), 2.62 (s, 1H, OH); ^{13}C {^1H} NMR: ($CDCl_3$, 100 MHz): δ 138.5, 138.0, 137.9, 131.1, 131.0, 128.4, 128.3, 127.9, 127.8, 127.7, 127.6, 127.5, 127.4, 126.6, 125.4 (Ar), 80.8 (C-3), 80.1 (C-4), 75.7 (CH_2Ph), 74.8 (CH_2Ph), 73.5 (CH_2Ph), 73.1 (C-5), 73.0 (C-2), 69.2 (C-10b), 68.9 (C-1'), 44.3 (C-4a); HRMS (ESI-TOF) *m/z*: $[M+H]^+$ calcd for $C_{34}H_{34}O_5S$ 555.2205; Found: 555.2203.

(2*R*,3*S*,4*R*,4a*S*,5*R*,10b*S*)-3,4-bis(benzyloxy)-2-[(benzyloxy)methyl]-9-methyl-2,3,4,4a,5,10b-hexahydrothiochromeno[4,3-*b*]pyran-5-ol (3b): 71% yield, light yellow crystals, mp 110–113 °C, $[\alpha]_D$ (*c* 0.1, $CHCl_3$) +15.80; IR (neat cm^{-1}) 3334, 3026, 2851, 1496, 1354, 695; ^1H NMR: ($CDCl_3$, 400 MHz): δ 7.45-7.21 (m , 16H, Ar), 7.13-7.05 (m, 2H, Ar), 6.94 (s, 1H, Ar), 5.48 (d, *J* = 4 Hz, 1H, H-5), 5.29 (d, *J* = 5.6 Hz, 1H, H-10b), 4.89 (d, *J* = 10.8 Hz, 1H, CH_AH_BPh), 4.78-4.67 (m, 3H, CH_AH_BPh, 2x CH_AH_BPh), 4.56 (d, *J* = 12 Hz,

1H, $CH_A\textit{H}_B$Ph), 4.50 (d, *J* = 10.8 Hz, CH_AH_BPh), 3.79-3.64 (m, 4H, H-1`a, H-1`b, H-3 and H-4), 3.57-3.51 (m, 1H, H-2), 2.75-2.66 (m, 1H, H-4a), 2.28 (d, *J* = 4.8 Hz, 1H, OH), 2.25 (s, 3H, CH_3); $^{13}C\{^1H\}$ NMR: ($CDCl_3$, 100 MHz): δ 138.89, 137.9, 135.5, 130.8, 128.9, 128.5, 128.4, 127.9, 127.8, 127.7, 127.6, 127.4, 126.6, (Ar), 81.1 (C-3), 80.3 (C-4), 75.8 (CH_2Ph), 74.8 (CH_2Ph), 73.5 (CH_2Ph), 73.2 (C-5,C-2), 69.3 (C-10b, C-1`), 44.3 (C-4a), 21.0 (CH_3); HRMS (ESI-TOF) *m/z*: $[M+H]^+$ calcd for $C_{35}H_{37}O_5S$ 569.2356, Found 569.2347.

(2*R*,3*S*,4*R*,4a*S*,5*R*,10b*S*)-3,4-bis(benzyloxy)-2-[(benzyloxy)methyl]-9-methoxy-2,3,4,4a,5,10b-hexahydrothiochromeno[4,3-*b*]pyran-5-ol (3c): 73%; yellowish crystals; mp 96-100 °C; $[\alpha]_D$ (*c* 0.1, $CHCl_3$) +14.0; IR (neat cm^{-1}) 3304, 3004, 2914, 1563, 1454, 1229, 696; ^1H NMR: ($CDCl_3$, 400 MHz): *δ* 7.41-7.21 (m, 15H, Ar), 7.13-7.07 (m, 2H, Ar), 6.95 (d, *J* = 8.8 Hz, 1H, Ar), 6.75 (dd, *J* = 2.4 Hz and *J* = 8.4 Hz, 1H, Ar), 5.47 (d, *J* = 4.0 Hz, 1H, H-10b), 5.29 (d, *J* = 6.0 Hz, 1H, H-5), 4.90 (d, *J* = 10.8 Hz, 1H, $CH_A\textit{H}_B$Ph), 4.76 (d, *J* = 6.0 Hz, 1H, $C\textit{H}_AH_B$Ph), 4.70 (d, *J* = 10.8 Hz, 1H, $CH_A\textit{H}_B$Ph), 4.67 (d, *J* = 6.0 Hz, 1H, $C\textit{H}_AH_B$Ph), 4.56 (d, *J* = 12.0 Hz, 1H, CH_AH_BPh), 4.51 (d, *J* = 10.8 Hz, 1H, CH_AH_BPh), 3.75 (d, *J* = 3.6 Hz, 1H, H-4), 3.72 (d, *J* = 9.6 Hz, H-3), 3.69-3.65 (m, 5H, H-1`a, H-1`b and OCH_3), 3.59-3.53 (m, 1H, H-2), 2.74-2.65 (m, 1H, H-4a), 2.46 (s, 1H, OH); $^{13}C\{^1H\}$ NMR: ($CDCl_3$, 100 MHz): *δ* 158.0, 138.5, 138.0, 137.9, 132.2, 128.5, 128.3, 128.0, 127.7, 127.6, 121.5, 115.5, 111.8, (Ar), 80.9 (C-3), 80.2 (C-4), 75.8 (CH_2Ph), 74.8 (CH_2Ph), 73.6 (CH_2Ph), 73.2 (C-5,C-2), 69.4 (C-10b), 69.2 (C-1`), 55.3 (OCH_3), 44.2 (C-4a); HRMS (ESI-TOF) *m/z*: $[M+H]^+$ calcd for $C_{35}H_{40}NO_6S^+$ 602.2571, Found 602.2568.

(2*R*,3*S*,4*R*,4a*S*,5*R*,10b*S*)-3,4-bis(benzyloxy)-2-[(benzyloxy)methyl]-9-(*tert*-butyl)-2,3,4,4a,5,10b-hexahydrothiochromeno[4,3-*b*]pyran-5-ol (3d): 80%; cream white crystals; mp 126-130 °C, $[\alpha]_D$ (*c* 0.1, $CHCl_3$) +38.0; IR (neat cm^{-1}) 3566, 3036, 2901, 1454, 1361, 697; ^1H NMR: ($CDCl_3$, 400 MHz): *δ* 7.68 (s, 1H, Ar), 7.48-7.19 (m, 14H, Ar), 7.16 (d, *J* = 8.4 Hz,

1H, Ar), 7.04 (s, 2H, Ar), 6.97 (d, J = 8.0Hz, 1H, Ar), 5.48 (t, J = 6.4 Hz, 1H, H-10b), 5.34 (d, J = 6.0 Hz, 1H, H-5), 4.91 (d, J = 11.2 Hz, 1H, CH_AH_BPh), 4.71 (dd, J = 9.6Hz and 11.6 Hz, 3H, CH_AH_BPh, 2 x CH_AH_BPh), 4.58 (d, J = 12.0 Hz, 1H, CH_AH_BPh), 4.45 (d, J = 10.4 Hz, CH_AH_BPh), 3.77-3.64 (m, 4H, H-1`a, H-1`b, H-3 and H-4), 3.52 (d, J = 9.2 Hz, 1H, H-2), 2.74-2.67 (m, 1H, H-4a), 2.30 (d, J = 2.4 Hz, 1H, OH), 1.25 (s, 9H, $C(CH_3)_3$); $^{13}C\{^1H\}$ NMR: ($CDCl_3$, 100 MHz): δ 148.73, 138.5, 137.9, 137.7, 130.3, 128.5, 128.4, 128.0, 127.9, 127.8, 127.7, 127.6, 127.4, 126.3, 125.1, 124.5, (Ar), 80.9 (C-3), 80.3 (C-4), 75.7 (CH_2Ph), 74.98 (CH_2Ph), 73.6 (CH_2Ph), 73.1 (C-5,C-2), 69.4 (C-10b), 69.0 (C-1`), 44.4 (C-4a), 34.5,31.2 ($C(CH_3)_3$); HRMS (ESI-TOF) *m/z*: $[M+H]^+$ calcd for $C_{38}H_{43}O_6S^+$ 611.2826, Found 611.2825.

General procedure for the synthesis of 2-*C*-hydroxymethyl glucosides 4a–d

To a solution of the hemithioacetal **3** (0.26 mmol) and nickel chloride hexahydrate (2.6 mmol) in a mixture of methanol (11 mL) and tetrahydrofuran (4 mL) at 0 °C was added sodium borohydride (7.8 mmol) in portions. After 10–30 min, the reaction mixture was filtered through a Celite® bed and the filtrate was dried *in vacuo*. The crude product was purified by column chromatography using ethylacetate:hexane (1:9) mixture as eluent to provide the corresponding glucosides **4a–d**:

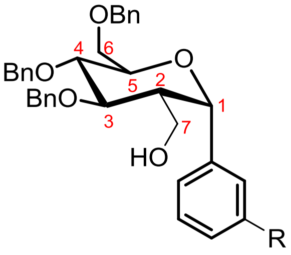

Phenyl 3,4,6-tri-*O*-benzyl-2-deoxy-2-*C*-hydroxymethyl-α-D-glucopyranoside (4a): 80% yield; colorless oil; $[\alpha]_D$ (*c* 0.1, $CHCl_3$) +2.0; IR (neat cm^{-1}) 3464, 2862, 1496, 1453, 1070,

1027, 734, 696; ^{1}H NMR (400 MHz, $CDCl_3$): δ 7.40–7.15 (m, 20H, Ar), 5.10 (d, *J* = 3.6 Hz, 1H, H-1), 4.68–4.49 (m, 6H, 3 x CH_2Ph), 4.25–4.16 (m, 1H, H-5), 4.02 (t, *J* = 4.4 Hz, 1H, H-3), 3.83 (dd, *J* = 6.0 and 10.0 Hz, 1H, H-6_a), 3.78–3.68 (m, 3H, H-4, H-6_b, H-7_a), 3.53–3.44 (m, 1H, H-7_b), 2.40–2.22 (m, 1H, H-2), 1.79 (bs, 1H, OH); $^{13}C\{^{1}H\}$ NMR (100 MHz, $CDCl_3$): δ 139.8, 138.2, 138.1, 137.7, 128.5, 128.3, 128.2, 127.8, 127.7, 127.6, 127.5, 127.2, 126.2 (Ar), 76.9 (C-3), 74.6 (C-5), 74.3 (C-4), 73.2 (CH_2Ph), 72.5 (CH_2Ph), 72.4 (CH_2Ph), 70.7 (C-1), 68.3 (C-6), 60.4 (C-7), 45.6 (C-2); HRMS (ESI-TOF) *m/z*: $[M+H]^+$ calcd for $C_{34}H_{37}O_5$ 525.2641; Found 525.2648.

3-Methylphenyl 3,4,6-tri-*O*-benzyl-2-deoxy-2-*C*-hydroxymethyl-α-D-glucopyranoside (4b):

84% yield; colorless oil; $[\alpha]_D$ (*c* 0.1, $CHCl_3$) +4.6; IR (neat cm^{-1}) 344, 2862, 1495, 1453, 1068, 1026, 734, 696; ^{1}H NMR (400 MHz, $CDCl_3$): δ 7.36-7.21 (m, 15H, Ar), 7.19 (d, *J* = 7.6 Hz, 1H, Ar), 7.16-7.10 (m, 2H, Ar), 7.05 (d, *J* = 7.6 Hz, 1H, Ar), 5.08 (d, *J* = 3.6 Hz, 1H, H-1), 4.65 (d, *J* = 11.6 Hz, 1H, $C\mathit{H}_AH_BPh$), 4.64 (d, *J* = 11.6 Hz, 1H, $C\mathit{H}_AH_BPh$), 4.60 (d, *J* = 11.6 Hz, 1H, $CH_A\mathit{H}_BPh$), 4.60 (d, *J* = 11.6 Hz, 1H, $CH_A\mathit{H}_BPh$), 4.54 (d, *J* = 12.0 Hz, 1H, $C\mathit{H}_AH_BPh$), 4.48 (d, *J* = 12.0 Hz, 1H, $CH_A\mathit{H}_BPh$), 4.22 (dd, *J* = 5.6 and 9.2 Hz, 1H, H-5), 4.02 (t, *J* = 4.4 Hz, 1H, H-3), 3.84 (dd, *J* = 6.0 and 10.0 Hz, 1H, H-6a), 3.79-3.71 (m, 3H, H-4, H-6_b and H-7_a), 3.51 (dd, *J* = 5.6 and 11.6 Hz, 1H, H-7_b), 2.37-2.23 (m, 4H, H-2 and CH_3), 1.71 (bs, 1H, OH); $^{13}C\{^{1}H\}$ NMR (100 MHz, $CDCl_3$): δ 139.6, 138.1, 138.0, 137.7, 128.5, 128.3, 128.2, 128.0, 127.9, 127.8, 127.7, 127.6, 127.0, 123.3, 74.5, 74.3, 73.2, 72.5, 70.8, 68.2, 60.5, 45.6, 21.5; HRMS (ESI-TOF) *m/z*: $[M+H]^+$ calcd for $C_{35}H_{39}O_5$ 539.2797; Found 539.2795.

3-Methoxyphenyl 3,4,6-tri-*O*-benzyl-2-deoxy-2-*C*-hydroxymethyl-α-D-glucopyranoside (4c): 87%; colorless oil; $[\alpha]_D$ (*c* 0.1, $CHCl_3$) + 5.50; IR (neat cm^{-1}) 3476, 3030, 2923, 1747,

1601, 1492, 1455, 1436, 1367, 1256, 1069, 696, 601; ^{1}H NMR (400 MHz, $CDCl_3$): δ 7.38-7.19 (m, 16H, Ar), 6.94-6.86 (m, 2H, Ar), 6.78 (dd, *J* = 2.4 and 8.0 Hz, 1H, Ar), 5.08 (d, *J* = 3.6 Hz, 1H, H-1), 4.68-4.42 (m, 6H, 3 x CH_2Ph), 4.26-4.7 (m, 1H, H-5), 4.02 (t, *J* = 4.4 Hz, 1H, H-3), 3.87-3.70 (m, 7H, H-4, H-6_a, H-6_b, H-7_a and OCH_3), 3.50 (dd, *J* = 5.6 and 11.6 Hz, 1H, H-7_b), 2.38-2.21 (m, 1H, H-2), 1.74 (bs, 1H, OH); ^{13}C{^{1}H} NMR (100 MHz, $CDCl_3$): δ 159.7, 141.5, 138.2, 138.1, 137.7, 129.3, 128.5, 128.3, 127.8, 127.7, 127.6, 118.4, 112.8, 111.9, 74.6, 74.2, 73.2, 72.5 (x 2), 70.5, 68.3, 60.4, 55.2, 45.7; HRMS (ESI-TOF) *m/z*: $[M+H]^+$ calcd for $C_{35}H_{39}O_6$ 555.2747; Found 555.2741.

3-*tert*-Butylphenyl 3,4,6-tri-*O*-benzyl-2-deoxy-2-*C*-hydroxymethyl-α-D-glucopyranoside (4d): 83%; colorless oil; $[\alpha]_D$ (*c* 0.1, $CHCl_3$) +7.50; IR (neat cm^{-1}) 3464, 3030, 2952, 2867, 1604, 1585, 1495, 1364, 1027, 1071, 696, 616; ^{1}H NMR (400 MHz, $CDCl_3$): δ 7.43-7.11 (m, 19H, Ar), 5.11 (d, *J* = 3.6 Hz, 1H, H-1), 4.71-4.46 (m, 6H, 3 x CH_2Ph), 4.24-4.16 (m, 1H, H-5), 4.03 (t, *J* = 4.4 Hz, 1H, H-3), 3.89-3.71 (m, 4H, H-4, H-6_a, H-6_b, H-7_a), 3.52 (dd, *J* = 5.4 Hz, 1H, H-7_b), 2.39-2.21 (m, 1H, H-2), 1.78 (s, 1H, OH), 1.30 (s, 9H, $C(CH_3)_3$); ^{13}C{^{1}H} NMR (100 MHz, $CDCl_3$): δ 151.2, 139.3, 138.2, 138.1, 137.8, 128.5, 128.3, 127.9, 127.8, 127.7, 127.5, 124.2, 123.4, 123.3, 74.5 (x 2), 73.2, 72.6 (x 2), 71.2, 68.4, 60.6, 45.9, 34.7, 31.4; HRMS (ESI-TOF) *m/z*: $[M+H]^+$ calcd for $C_{38}H_{45}O_5$ 581.3267; Found 581.3266.

General procedure for the synthesis of 2-*C*-carbaldehyde glucosides 5a–d and mannosides 5a'–d'

To a solution of hemithioacetal **3** (0.18 mmol) in acetone (2 mL) was added freshly prepared W-1 Raney nickel (1 spatula) and the reaction mixture was stirred at room temperature for 45 min. The reaction mixture was then filtered through a Celite® bed and the filtrate was dried in

vacuo. The crude product was purified by column chromatography using ethylacetate:hexane (1:9) mixture as eluent to provide the corresponding carbaldehydes:

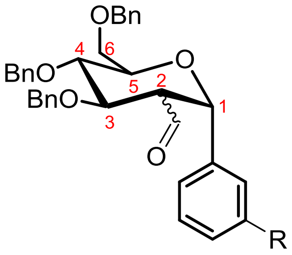

Phenyl 3,4,6-tri-*O*-benzyl-2-deoxy-2-*C*-carbaldehyde-α-D-glucopyranoside (5a) and phenyl 3,4,6-tri-*O*-benzyl-2-deoxy-2-*C*-carbaldehyde-α-D-mannopyranoside (5a'): 3:1 mixture of carbaldehyde **5a** and **5a'**; in 78% yield; colorless oil; IR (neat cm^{-1}): 2859, 1714, 1453, 1634, 1205, 1070,734, 696; ^{1}H NMR (400 MHz, $CDCl_3$) for **5a**: δ 9.58 (d, *J* = 2.4 Hz, 1H, CHO), 7.60–7.10 (m, 20H, Ar), 5.10 (d, *J* = 2.8 Hz, 1H, H-1), 4.54-4.33 (m, 5H, 2 x CH_2Ph and CH_AH_BPh), 4.29 (d, *J* = 12.0 Hz, 1H, CH_AH_BPh), 4.23 (dt, *J* = 2.2 and 6.8 Hz, 1H, H-5), 4.01 (t, *J* = 3.8 Hz, 1H, H-3), 3.85 (dd, *J* = 6.8 and 10.4 Hz, 1H, H-6_a), 3.72-3.61 (m, 1H, H-6_b). 3.52 (dd, *J* = 2.2 and 3.8 Hz, 1H, 1H, H-4), 2.71 (dd, *J* = 2.8 and 6.8 Hz, 1H, H-2); ^{13}C{^{1}H} NMR (100 MHz, $CDCl_3$) for **5a**: δ 201.2 (CHO), 139.0, 138.1, 137.6, 137.4, 128.6, 128.5, 128.4, 128.3, 128.0, 127.8, 125.9 (Ar), 75.9 (C-3), 75.5 (C-5), 73.1 (CH_2Ph), 72.6 (C-4), 72.5 (CH_2Ph), 71.6 (CH_2Ph), 67.5 (C-1), 67.4 (C-6), 53.3 (C-2). ^{1}H NMR (400 MHz, $CDCl_3$) for **5a'**: δ 9.79 (d, *J* = 2.0 Hz, 1H, CHO), 5.21 (d, *J* = 5.2 Hz, 1H, H-1), 4.61 (d, *J* = 11.2 Hz, 1H, CH_AH_BPh), 3.93 (dd, *J* = 4.4 and 6.8 Hz, 1H, H-3), 3.78 (t, J = 6.8 Hz, 1H, H-4) 3.16 (dt, *J* = 2.0 and 5.2 Hz, 1H, H-2); ^{13}C{^{1}H} NMR (100 MHz, $CDCl_3$) for **5a'**: δ 202.0 (CHO), 138.1, 138.0, 137.9, 137.5, 128.5, 128.2, 128.1, 127.8, 127.7, 127.4, 126.7 (Ar), 76.3 (C-3), 74.0 (C-4), 73.8 (CH_2Ph), 73.5 (CH_2Ph), 73.3 (CH_2Ph), 71.8 (C-1), 68.6 (C-6), 52.9 (C-2). HRMS (ESI-TOF) *m/z*: $[M+NH_4]^+$ calcd for $C_{34}H_{38}NO_5$ 540.2750; Found 540.2747.

3-Methylphenyl 3,4,6-tri-*O*-benzyl-2-deoxy-2-*C*-carbaldehyde-α-D-glucopyranoside (5b) and 3-methylphenyl 3,4,6-tri-*O*-benzyl-2-deoxy-2-*C*-carbaldehyde-α-D-mannopyranoside (5b'): >25:1 mixture of carbaldehyde **5b** and **5b'**; 80% yield; colorless oil; $[\alpha]_D$ (*c* 0.1, $CHCl_3$) +12.1; IR (neat cm^{-1}) 2862, 1712, 1453, 1363, 1068, 734, 696; ^1H NMR (400 MHz, $CDCl_3$) δ 9.63 (d, *J* = 1.6 Hz, 1H, CHO), 7.31-7.12 (m, 16H, Ar), 7.07 (bs, 1H, Ar), 7.00 (dd, *J* = 7.6 and 16.0 Hz, 2H, Ar), 5.12 (s, 1H, H-1), 4.61-4.24 (m, 7H, 3 x CH_2Ph and H-5), 4.09-4.02 (m, 1H, H-3), 3.90 (dd, *J* = 7.2 and 10.0 Hz, H-6_a), 3.70 (dd, *J* = 6.0 and 10.0 Hz, 1H, H-6_b), 3.59-3.53 (m, 1H, H-4), 2.74 (bs, 1H, H-2), 2.25 (s, 3H, CH_3); $^{13}C\{^1H\}$ NMR (100 MHz, $CDCl_3$) δ 201.4, 138.9, 138.2, 138.1, 137.6, 137.3, 128.6, 128.4, 128.3, 128.1, 128.0, 127.8, 127.6, 126.6, 122.9, 75.9, 75.5, 73.1, 72.6, 72.4, 71.5, 67.4, 67.3, 53.1, 21.5. HRMS (ESI-TOF) *m/z*: $[M+NH_4]^+$ calcd for $C_{35}H_{40}NO_5$ 554.2906; Found 554.2926.

3-Methoxyphenyl 3,4,6-tri-*O*-benzyl-2-deoxy-2-*C*-carbaldehyde-α-D-glucopyranoside (5c) and 3-methoxyphenyl 3,4,6-tri-*O*-benzyl-2-deoxy-2-*C*-carbaldehyde-α-D-mannopyranoside (5c'): 1:13 mixture of carbaldehyde **5c** and **5c'**; in 84% yield; colorless oil; $[\alpha]_D$ (*c* 0.1, $CHCl_3$) +14.5; IR (neat cm^{-1}) 2862, 1714, 1600, 1585, 1491, 1453, 1262, 1069, 735, 696; ^1H NMR (400 MHz, $CDCl_3$) for **5c**: δ 9.70 (d, *J* = 2.4 Hz, 1H, CHO), 7.38-7.16 (m, 16H, Ar), 6.93 (d, *J* = 7.2 Hz, 1H, Ar), 6.84 (d, *J* = 7.6 Hz, 1H, Ar), 6.78 (dt, *J* = 2.4 and 6.0 Hz, 1H, Ar) 5.19 (d, *J* = 2.4 Hz, 1H, H-1), 4.69-4.47 (m, 5H, CH_2Ph), 4.40 (d, *J* = 11.6 Hz, 1H, CH_AH_BPh), 4.37-4.32 (m, 1H, H-5), 4.12 (t, *J* = 3.8 Hz, 1H, H-3), 3.96 (dd, *J* = 6.8 and 10.0 Hz, 1H, H-6_a), 3.81-3.75 (m, 4H, H-6_b and OCH_3), 3.64 (t, 3.0 Hz, 1H, H-4), 2.83-2.77 (m, 1H, H-2);); $^{13}C\{^1H\}$ NMR (100 MHz, $CDCl_3$) for **5c**: δ 201.1, 159.9, 140.8, 138.1, 137.6, 137.4, 129.5, 128.6, 128.5, 128.4, 128.3, 128.2, 128.0, 127.9, 127.8, 127.7, 127.6, 118.1, 113.1, 111.6, 76.0, 75.6, 73.1, 72.6, 72.5, 71.6, 67.4 (x2), 55.3, 53.3; ^1H NMR (400 MHz, $CDCl_3$) for **5c'**: δ 9.93 (d, *J* = 1.6 Hz, 1H, CHO), 5.31 (d, *J* = 4.8 Hz, 1H, H-1),

4.73 (d, J = 11.2 Hz, 1H, CH_AH_BPh), 4.08-4.02 (m, 1H, H-3), 3.27 (dt, J = 1.6 and 4.4 Hz, 1H, H-2); HRMS (ESI-TOF) *m/z*: $[M+NH_4]^+$ calcd for $C_{35}H_{40}NO_6$ 570.2856; Found 570.2853.

3-*tert*-Butylphenyl 3,4,6-tri-*O*-benzyl-2-deoxy-2-*C*-carbaldehyde-α-D-glucopyranoside (5d) and 3-*tert*-butylphenyl 3,4,6-tri-*O*-benzyl-2-deoxy-2-*C*-carbaldehyde-α-D-mannopyranoside (5d): 1:8 mixture of carbaldehyde **5d** and **5d'**; colorless oil; $[\alpha]_D$ (*c* 0.1, $CHCl_3$) +16.3; IR (neat cm^{-1}) 2864, 1687, 1491, 1453, 1363, 1073, 734, 697; ^{1}H NMR (400 MHz, $CDCl_3$) for **5d** δ 9.71 (d, J = 2.0 Hz, 1H, CHO), 7.40-7.8 (m, 18H, Ar), 7.12 (d, J = 6.4 Hz, 1H, Ar), 5.22 (d, J = 2.0 Hz, 1H, H-1), 4.70-4.46 (m, 5H, CH_2Ph), 4.41 (d, J = 11.6 Hz, 1H, CH_AH_BPh), 4.38-4.29 (m, 1H, H-5), 4.13 (t, J = 4.0 Hz, 1H, H-3), 3.96 (dd, J = 6.6 and 10.4 Hz, 1H, H-6_a), 3.80 (dd, J = 6.0 and 10.4 Hz, 1H, H-6_b), 3.66 (t, J = 3.2 Hz, 1H, H-4), 2.82 (bd, J = 3.2 Hz, 1H, H-2), 1.29 (s, 9H, $C(CH_3)_3$); $^{13}C\{^1H\}$ NMR (100 MHz, $CDCl_3$) for **5d** δ 201.3, 151.5, 138.7, 138.2, 137.7, 137.5, 128.6, 128.5, 128.3, 128.2, 128.1, 128.0, 127.8, 127.7, 127.6, 124.4, 123.2, 122.9, 76.2, 75.5, 73.1, 72.8, 72.7, 71.7, 68.0, 67.6, 53.5, 34.7, 31.4; ^{1}H NMR (400 MHz, $CDCl_3$) for **5d'**: δ 9.94 (d, J = 1.6 Hz, 1H, CHO), 6.96 (d, J = 7.6 Hz, 1H, Ar), 3.41-3.32 (m, 1H, H-2), 1.26 (s, 9H, $C(CH_3)_3$); HRMS (ESI-TOF) *m/z*: $[M+NH_4]^+$ calcd for $C_{38}H_{46}NO_5$ 596.3376; Found 596.3381.

General procedure for the synthesis of 2,3-unsaturated carbaldehydes 9a–c

To a solution of 2-*C*-carbaldehyde **5** (0.19 mmol) in methanol (3 mL) was added a catalytic amount of K_2CO_3 and the resulting reaction mixture was stirred for 30 min at room temperature. After completion of the reaction, the reaction mixture was diluted with water (5 mL). The solution was then extracted with ethyl acetate (3 × 5 mL). The combined organic layers were dried over $MgSO_4$, filtered and the filtrate was evaporated to dryness in vacuo.

The crude product was purified by flash column chromatography using ethyl acetate:hexane (1:9) mixture as eluent to provide the corresponding 2,3-unsatured carbaldehydes **9a–c**:

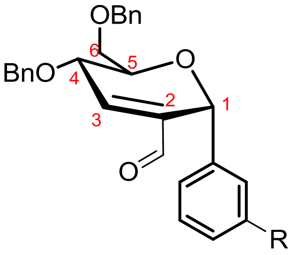

1-Phenyl-2,3-dideoxy-*C*-2-formyl-4,6-di-*O*-benzyl-1,5-anhydro-D-*arabino*-hex-2-enitol (9a): 98% yield; colorless oil; $[\alpha]_D$ (*c* 0.1, $CHCl_3$) +15.6; IR (neat cm^{-1}) 2856, 1686, 1495, 1453, 1179, 1072, 869, 733, 696; ^{1}H NMR (400 MHz, $CDCl_3$) δ 9.46 (s, 1H, CHO), 7.31-7.17 (m, 1H, Ar), 7.01 (s, 1H, H-3), 5.57 (s, 1H, H-1), 4.59 (d, J = 11.6 Hz, 1H, CH_AH_BPh ($C\mathit{H}_A H_B$Ph)), 4.50 (d, J = 12.0 Hz, 1H, $C\mathit{H}_A H_B$Ph), 4.48-4.43 (m, 2H, $CH_A\mathit{H}_B$Ph and H-4), 4.37 (d, J = 12.0 Hz, 1H, $CH_A\mathit{H}_B$Ph), 3.57 (dd, J = 3.2 and 10.4 Hz, 1H, H-6_a), 3.53-3.42 (m, 2H, H-5 and H-6_b); $^{13}C\{^1H\}$ NMR (100 MHz, $CDCl_3$) δ 191.0 (CHO), 147.9, 141.8, 137.7, 137.3, 128.7, 128.5, 128.4, 128.3, 128.2, 128.1, 128.0, 127.7, 127.6, 126.9, 73.3, 73.2, 72.3, 70.3, 69.5, 68.3; HRMS (ESI-TOF) *m/z*: $[M+NH_4]^+$ calcd for $C_{27}H_{30}NO_4$ 432.2175; Found 432.2167.

1-(*m*-Tolyl)-2,3-dideoxy-*C*-2-formyl-4,6-di-*O*-benzyl-1,5-anhydro-D-*arabino*-hex-2-enitol (9b): 95% yield; colorless oil; $[\alpha]_D$ (*c* 0.1, $CHCl_3$) +14.3; IR (neat cm^{-1}) 2860, 1686, 1495, 1453, 1179, 1072, 869, 734, 697; ^{1}H NMR (400 MHz, $CDCl_3$) δ 9.50 (s, 1H, CHO), 7.37-7.13 (m, 11H, Ar), 7.11-7.01 (m, 4H, H-3 and Ar), 5.57 (s, 1H, 5.57 (s, 1H, H-1), 4.64 (d, J = 10.8 Hz, 1H, $C\mathit{H}_A H_B$Ph), 4.55 (d, J = 11.6 Hz, 1H, $C\mathit{H}_A H_B$Ph), 4.51-4.45 (m, 2H, H-4 and $CH_A\mathit{H}_B$Ph), 4.37 (d, J = 12.0 Hz, 1H, $CH_A\mathit{H}_B$Ph), 3.64-3.53 (m, 2H, H-5 and H-6_a), 3.51 (d, J = 10.4 Hz, 1H, H-6_b), 2.29 (s, 3H, CH_3); $^{13}C\{^1H\}$ NMR (100 MHz, $CDCl_3$) δ 191.0, 147.6, 141.9, 138.0, 137.8, 137.4, 137.2, 129.5, 129.2, 128.5, 128.3, 128.2, 128.1, 128.0, 127.7,

127.6, 126.9, 125.7, 73.3, 73.2, 72.4, 70.5, 69.6, 68.5, 65.3, 21.4; HRMS (ESI-TOF) *m/z*: $[M+NH_4]^+$ calcd for $C_{28}H_{32}NO_4$ 446.2331; Found 446.2334.

1-(*m-tert*-Butylphenyl)-2,3-dideoxy-*C*-2-formyl-4,6-di-*O*-benzyl-1,5-anhydro-D-*arabino*-hex-2-enitol (9c): 94% yield; colorless oil; $[\alpha]_D$ (*c* 0.1, $CHCl_3$) +17.0; IR (neat cm^{-1}) 2856, 1686, 1495, 1453, 1179, 1072, 869, 734, 697; ^1H NMR (400 MHz, $CDCl_3$) δ 9.51 (s, 1H, CHO), 7.37 (s, 1H, Ar), 7.34-7.14 (m, 12H, Ar), 7.04 (s, 1H, H-3), 6.96 (d, *J* = 7.2 Hz, 1H, Ar), 5.61 (s, 1H, H-1), 4.63 (d, *J* = 11.6 Hz, 1H, CH_AH_BPh), 4.55 (d, *J* = 12.0 Hz, 1H, CH_AH_BPh), 4.53-4.43 (m, 2H, H-4 and CH_AH_BPh), 4.36 (d, *J* = 12.0 Hz, 1H, CH_AH_BPh), 3.64-3.55 (m, 2H, H-5 and H-6_a), 3.52 (d, *J* = 10.4 Hz, 1H, H-6_b), 1.26 (s, 9H, $C(CH_3)_3$); $^{13}C\{^1H\}$ NMR (100 MHz, $CDCl_3$) δ 191.0, 151.3, 147.6, 142.3, 137.8, 137.5, 137.0, 128.5, 128.3, 128.1, 128.0, 127.7, 126.0, 125.4, 73.4, 73.3, 72.2, 70.5, 69.6, 68.6, 34.7, 31.3; HRMS (ESI-TOF) *m/z*: $[M+NH_4]^+$ calcd for $C_{31}H_{38}NO_4$ 488.2801; Found 488.2809.

References

1. Perrin, D. D.; Armarego, L. F. *Purification of Laboratory Chemicals*, 3rd Ed.; Pergamon Press, Oxford, **1988**.

2. Kinfe, H. H.; Moshapo, P. T.; Makolo, F. L.; Muller, A. *J. Carbohydr. Chem.* **2013**, *32*, 139–157.

3. Kinfe, H.H.; Mebrahtu, F.M.; Makolo, F.L.; Moshapo, P.T.; Manana, M.M. *J. Org. Chem.* **2014**, *79*, 3111−3118.
