# Supplementary material for: Synthesis of 1,2-cis-2-C-branched aryl-C-glucosides via desulfurization of carbohydrate based hemithioacetals
Source: Beilstein J Org Chem. 2015 Apr 29;11:583–8. doi: 10.3762/bjoc.11.64 (PMC4464409; doi:10.3762/bjoc.11.64)
Supplement: File 2 — 1H and 13C NMR spectra. [file Beilstein_J_Org_Chem-11-583-s002.pdf]

Supporting Information

for

Synthesis of 1,2-*cis*-2-*C*-branched aryl-*C*-glucosides via desulfurization of carbohydrate based hemithioacetals

Henok H. Kinfe*, Fanuel M. Mebrahtu, Mandlenkosi M. Manana, Kagiso Madumo and Mokela S. Sokamisa

Address: Department of Chemistry, University of Johannesburg, PO Box 524, Auckland Park 2006, South Africa

*Corresponding author

Email: Henok H Kinfe - hhkinfe@uj.ac.za

^{1}H and ^{13}C NMR spectra

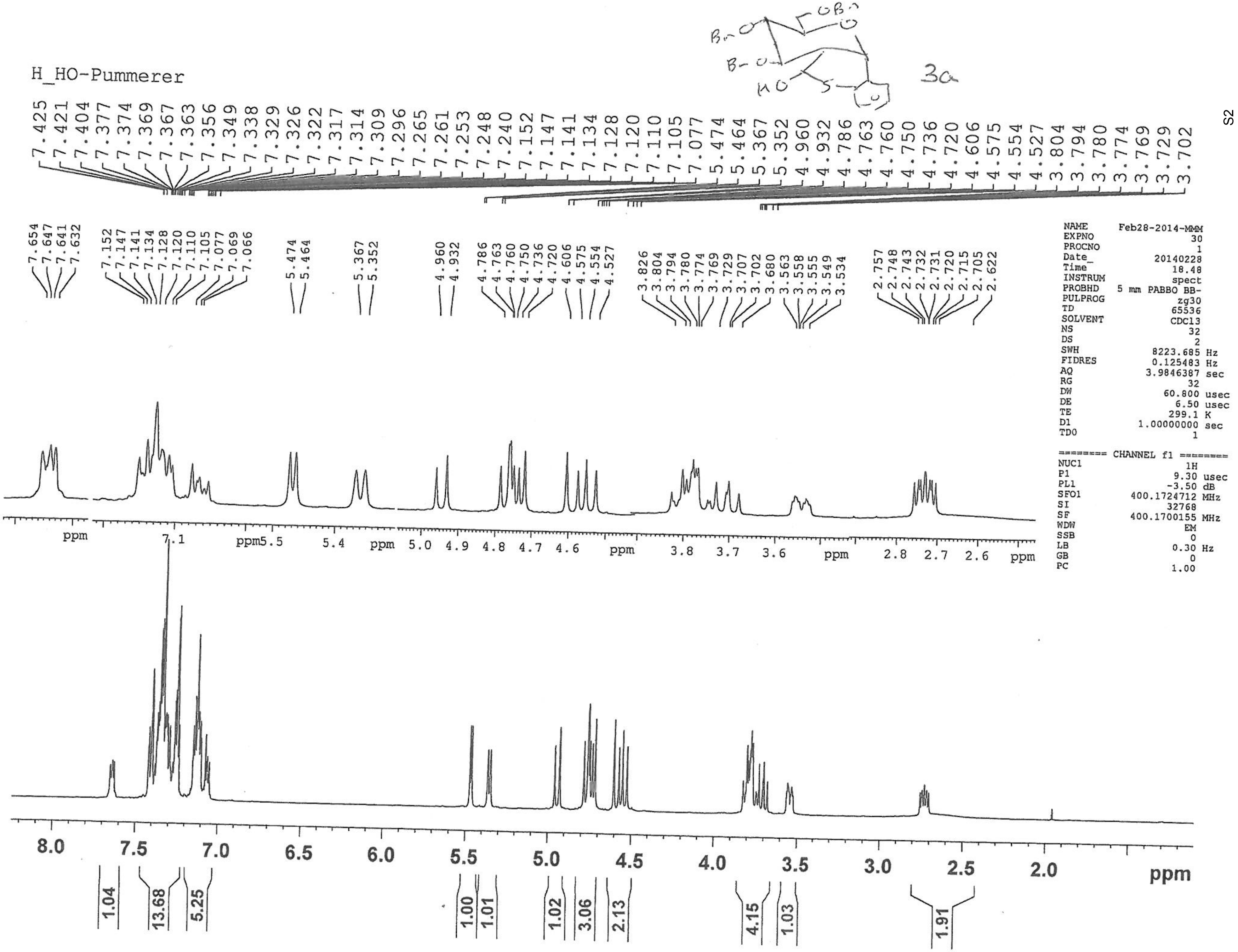
H_HO-Pummerer
3a
7.425 7.421 7.404 7.377 7.374 7.369 7.367 7.363 7.356 7.349 7.338 7.329 7.326 7.322 7.317 7.314 7.309 7.296 7.265 7.261 7.253 7.248 7.240 7.152 7.147 7.141 7.134 7.128 7.120 7.110 7.105 7.077 5.474 5.464 5.367 5.352 4.960 4.932 4.786 4.763 4.760 4.750 4.736 4.720 4.606 4.575 4.554 4.527 3.804 3.794 3.780 3.774 3.769 3.729 3.702
7.654 7.647 7.641 7.632
7.152 7.147 7.141 7.134 7.128 7.120 7.110 7.105 7.077 7.069 7.066
5.474 5.464
5.367 5.352
4.960 4.932
4.786 4.763 4.760 4.750 4.736 4.720 4.606 4.575 4.554 4.527
3.826 3.804 3.794 3.780 3.774 3.769 3.729 3.707 3.702 3.680 3.563 3.558 3.555 3.549 3.534
2.757 2.748 2.743 2.732 2.731 2.720 2.715 2.705 2.622
NAME Feb28-2014-MMM
EXPNO 30
PROCNO 1
Date_ 20140228
Time 18.48
INSTRUM spect
PROBHD 5 mm PABBO BB-
PULPROG zg30
TD 65536
SOLVENT CDCl3
NS 32
DS 2
SWH 8223.685 Hz
FIDRES 0.125483 Hz
AQ 3.9846387 sec
RG 32
DW 60.800 usec
DE 6.50 usec
TE 299.1 K
D1 1.00000000 sec
TD0 1
======== CHANNEL f1 ========
NUC1 1H
P1 9.30 usec
PL1 -3.50 dB
SFO1 400.1724712 MHz
SI 32768
SF 400.1700155 MHz
WDW EM
SSB 0
LB 0.30 Hz
GB 0
PC 1.00
ppm
7.1
ppm5.5
5.4
ppm 5.0 4.9 4.8 4.7 4.6
ppm
3.8 3.7 3.6
ppm
2.8 2.7 2.6
ppm
8.0 7.5 7.0 6.5 6.0 5.5 5.0 4.5 4.0 3.5 3.0 2.5 2.0
ppm
1.04 13.68 5.25 1.00 1.01 1.02 3.06 2.13 4.15 1.03 1.91

4-Me_HO-Pummerer

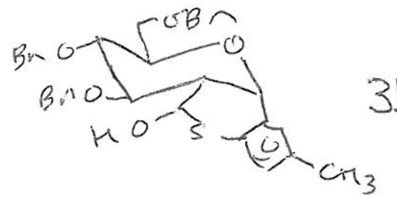

3b

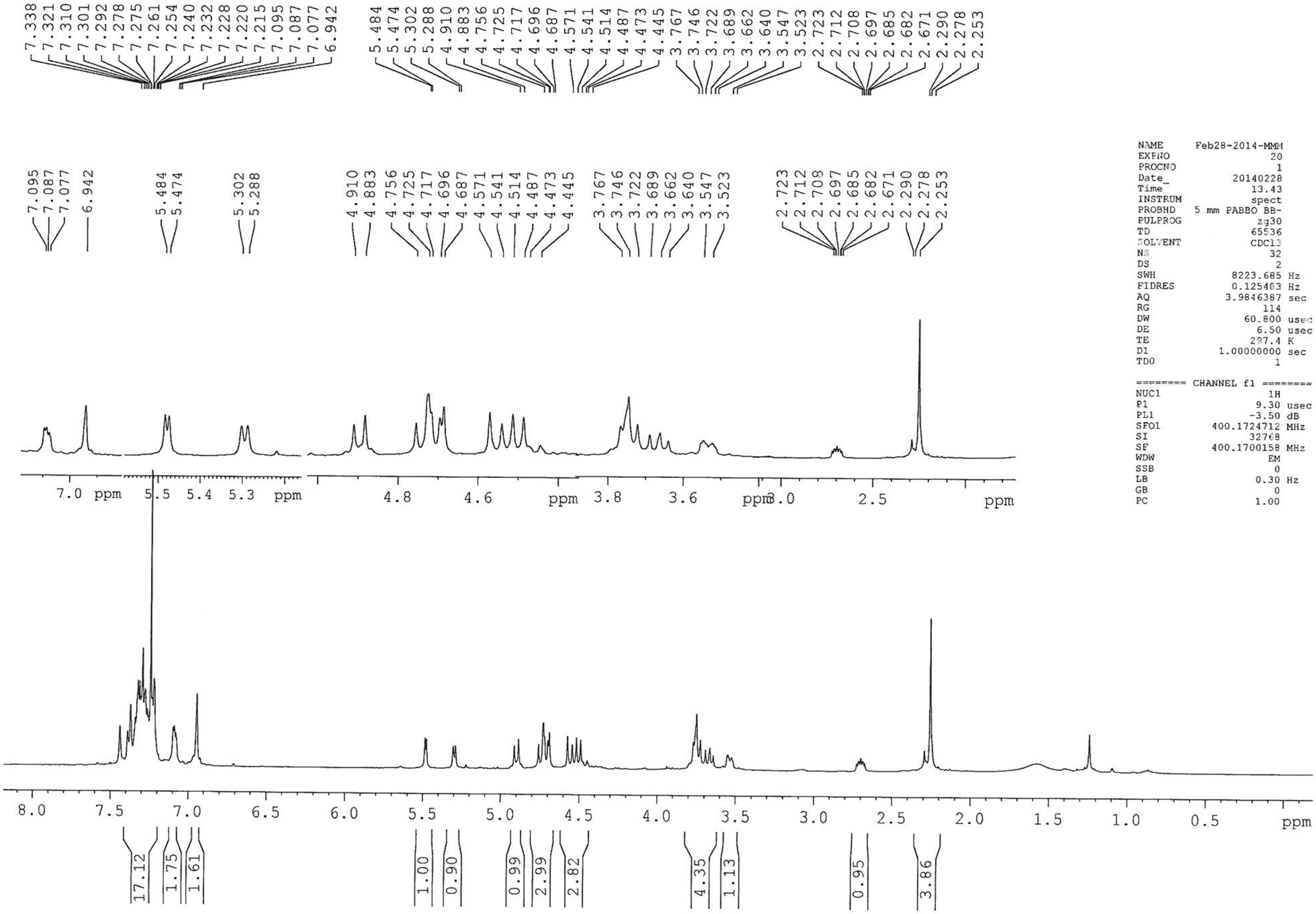

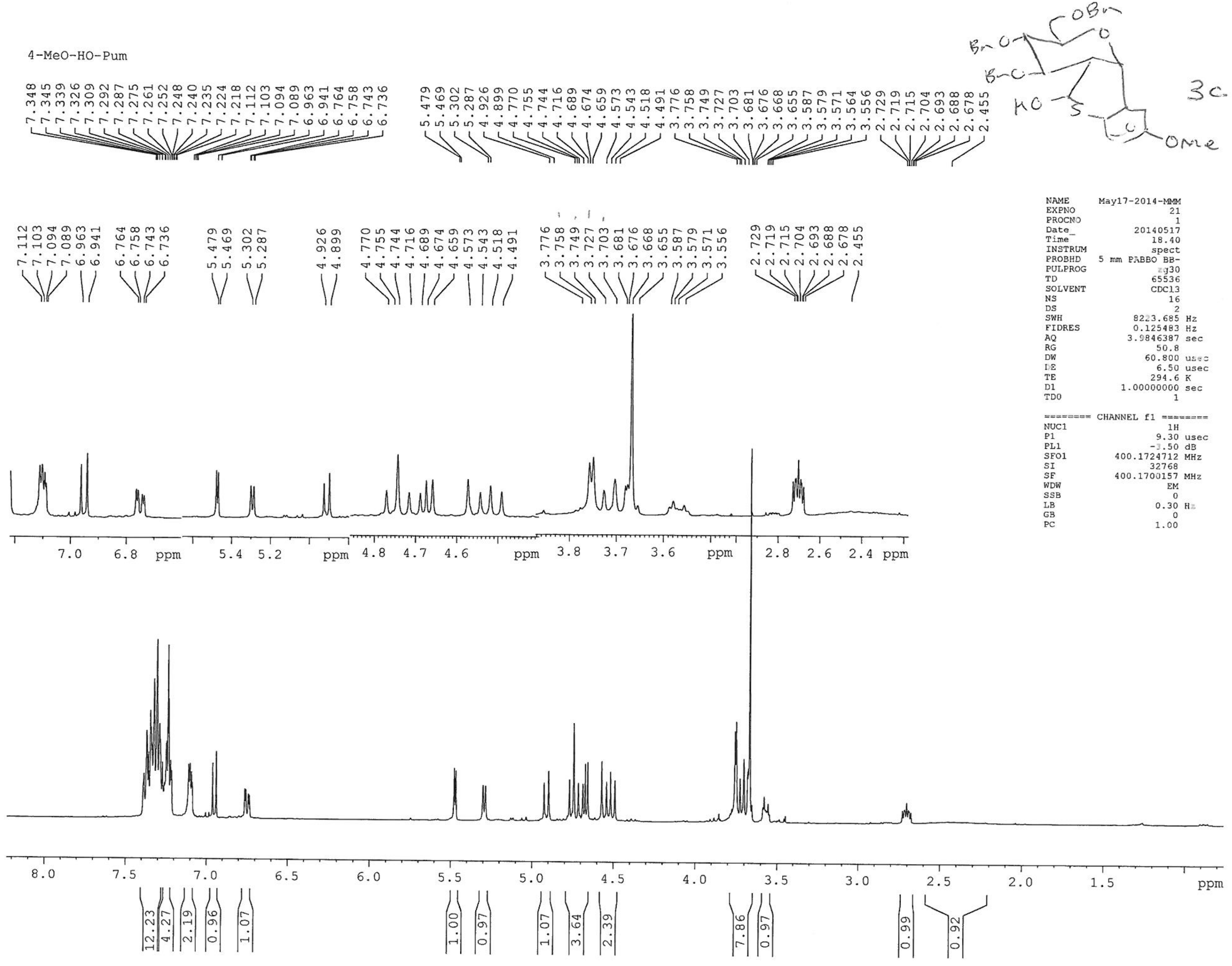
4-MeO-HO-Pum
3c
NAME May17-2014-MMM
EXPNO 21
PROCNO 1
Date_ 20140517
Time 18.40
INSTRUM spect
PROBHD 5 mm PABBO BB-
PULPROG zg30
TD 65536
SOLVENT CDCl3
NS 16
DS 2
SWH 8223.685 Hz
FIDRES 0.125483 Hz
AQ 3.9846387 sec
RG 50.8
DW 60.800 usec
DE 6.50 usec
TE 294.6 K
D1 1.00000000 sec
TD0 1
======== CHANNEL f1 ========
NUC1 1H
P1 9.30 usec
PL1 -3.50 dB
SFO1 400.1724712 MHz
SI 32768
SF 400.1700157 MHz
WDW EM
SSB 0
LB 0.30 Hz
GB 0
PC 1.00
ppm

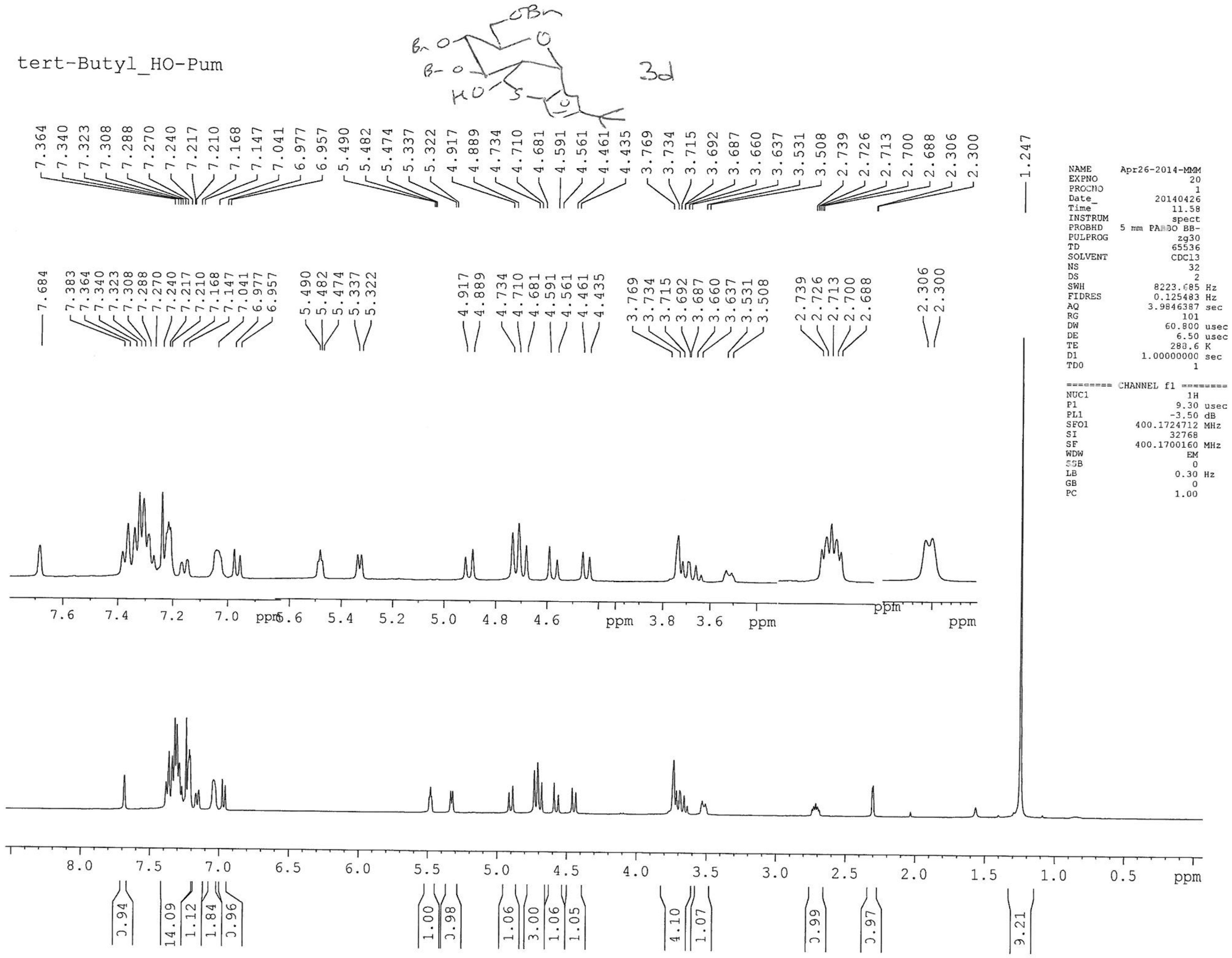
tert-Butyl_HO-Pum
3d
7.364 7.340 7.323 7.308 7.288 7.270 7.240 7.217 7.210 7.168 7.147 7.041 6.977 6.957 5.490 5.482 5.474 5.337 5.322 4.917 4.889 4.734 4.710 4.681 4.591 4.561 4.461 4.435 3.769 3.734 3.715 3.692 3.687 3.660 3.637 3.531 3.508 2.739 2.726 2.713 2.700 2.688 2.306 2.300 1.247
NAME Apr26-2014-MMM
EXPNO 20
PROCNO 1
Date_ 20140426
Time 11.58
INSTRUM spect
PROBHD 5 mm PABBO BB-
PULPROG zg30
TD 65536
SOLVENT CDCl3
NS 32
DS 2
SWH 8223.685 Hz
FIDRES 0.125483 Hz
AQ 3.9846387 sec
RG 101
DW 60.800 usec
DE 6.50 usec
TE 288.6 K
D1 1.00000000 sec
TD0 1
======== CHANNEL f1 ========
NUC1 1H
P1 9.30 usec
PL1 -3.50 dB
SFO1 400.1724712 MHz
SI 32768
SF 400.1700160 MHz
WDW EM
SSB 0
LB 0.30 Hz
GB 0
PC 1.00
ppm
0.94 14.09 1.12 1.84 0.96 1.00 0.98 1.06 3.00 1.06 1.05 4.10 1.07 0.99 0.97 9.21

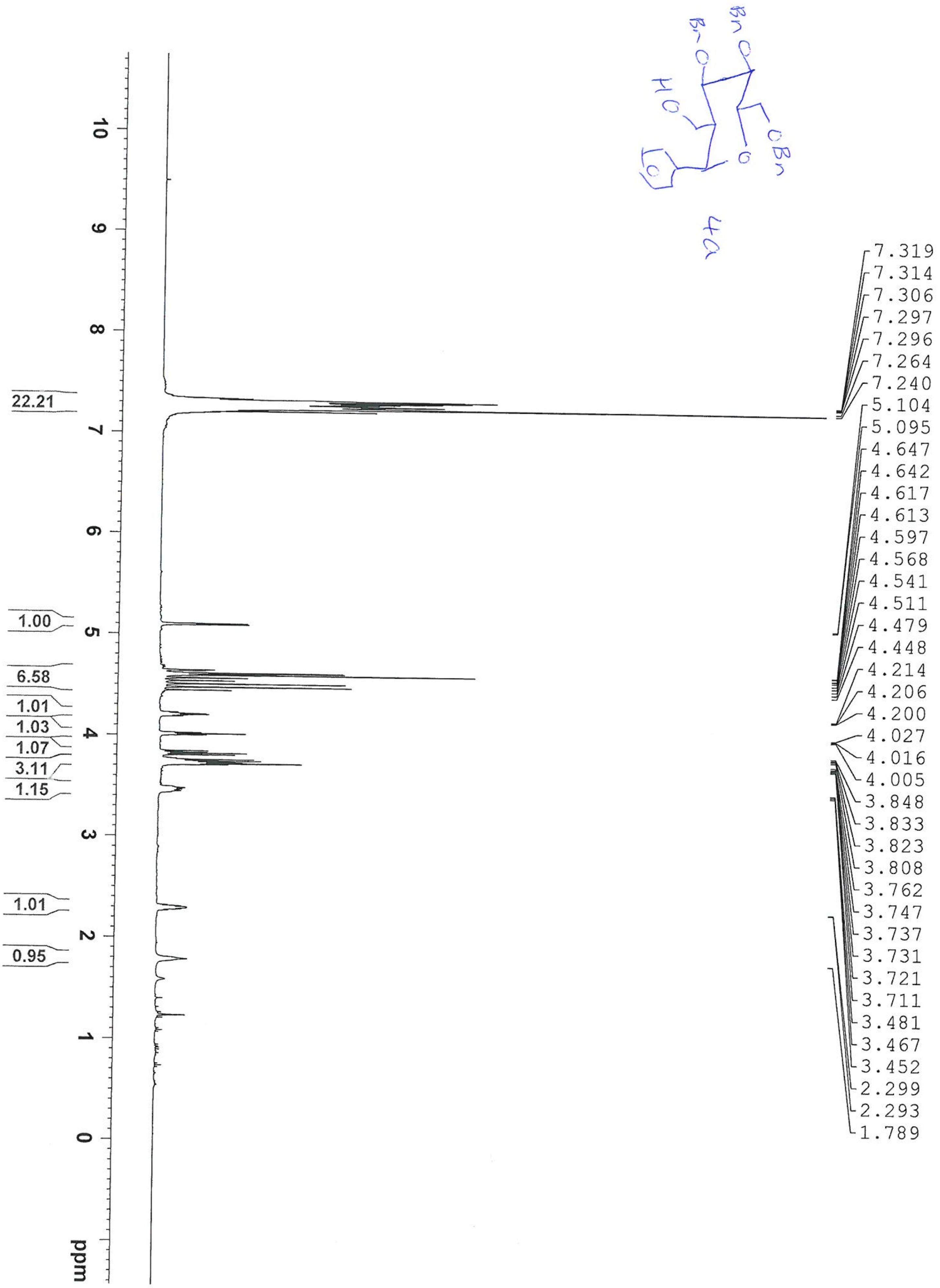
7.319
7.314
7.306
7.297
7.296
7.264
7.240
5.104
5.095
4.647
4.642
4.617
4.613
4.597
4.568
4.541
4.511
4.479
4.448
4.214
4.206
4.200
4.027
4.016
4.005
3.848
3.833
3.823
3.808
3.762
3.747
3.737
3.731
3.721
3.711
3.481
3.467
3.452
2.299
2.293
1.789
22.21
1.00
6.58
1.01
1.03
1.07
3.11
1.15
1.01
0.95
10
9
8
7
6
5
4
3
2
1
0
ppm
4a

4M_OH

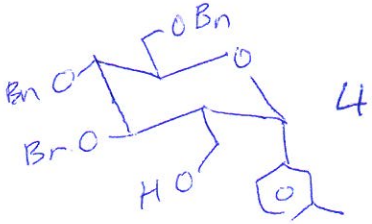

BnO
BnO
HO
OBn

4b

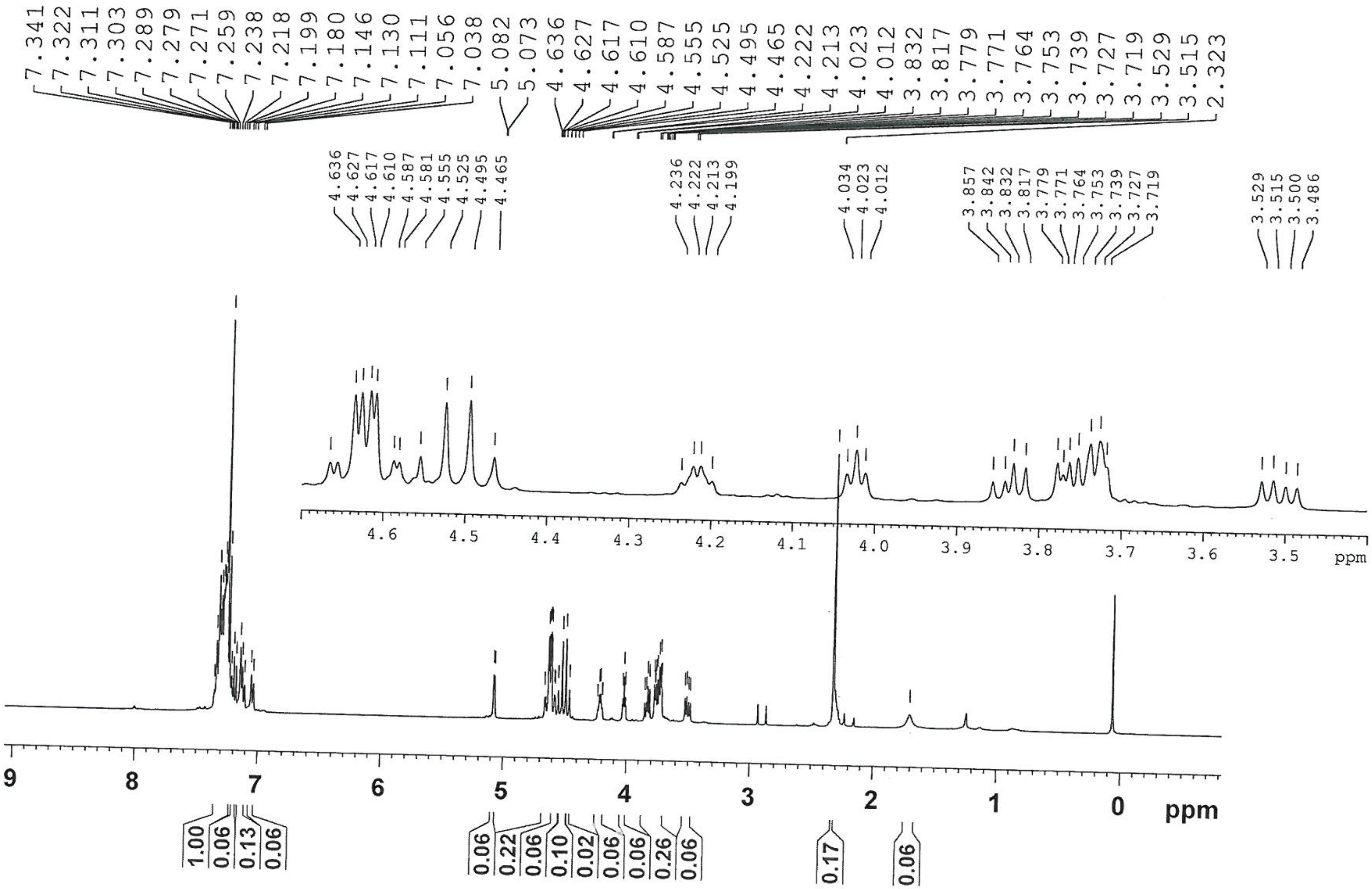

7.341
7.322
7.311
7.303
7.289
7.279
7.271
7.259
7.238
7.218
7.199
7.180
7.146
7.130
7.111
7.056
7.038
5.082
5.073
4.636
4.627
4.617
4.610
4.587
4.555
4.525
4.495
4.465
4.222
4.213
4.023
4.012
3.832
3.817
3.779
3.771
3.764
3.753
3.739
3.727
3.719
3.529
3.515
2.323
4.636
4.627
4.617
4.610
4.587
4.581
4.555
4.525
4.495
4.465
4.236
4.222
4.213
4.199
4.034
4.023
4.012
3.857
3.842
3.832
3.817
3.779
3.771
3.764
3.753
3.739
3.727
3.719
3.529
3.515
3.500
3.486
4.6
4.5
4.4
4.3
4.2
4.1
4.0
3.9
3.8
3.7
3.6
3.5
ppm
9
8
7
6
5
4
3
2
1
0
ppm
1.00
0.06
0.13
0.06
0.06
0.22
0.06
0.10
0.02
0.06
0.06
0.26
0.06
0.17
0.06

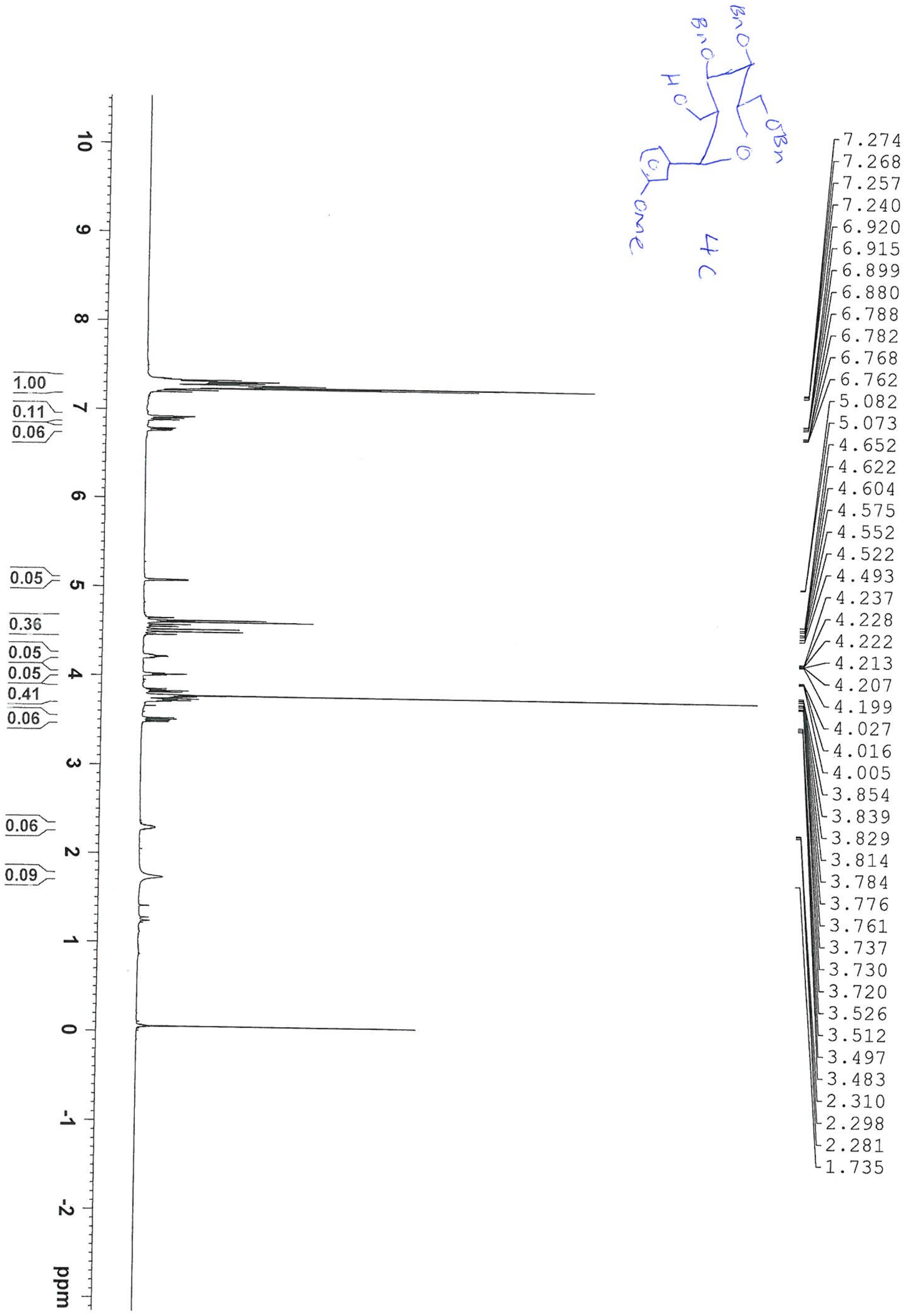
BnO
BnO
HO
OBn
O
OMe
4c
7.274
7.268
7.257
7.240
6.920
6.915
6.899
6.880
6.788
6.782
6.768
6.762
5.082
5.073
4.652
4.622
4.604
4.575
4.552
4.522
4.493
4.237
4.228
4.222
4.213
4.207
4.199
4.027
4.016
4.005
3.854
3.839
3.829
3.814
3.784
3.776
3.761
3.737
3.730
3.720
3.526
3.512
3.497
3.483
2.310
2.298
2.281
1.735
1.00
0.11
0.06
0.05
0.36
0.05
0.05
0.41
0.06
0.06
0.09
10
9
8
7
6
5
4
3
2
1
0
-1
-2
ppm

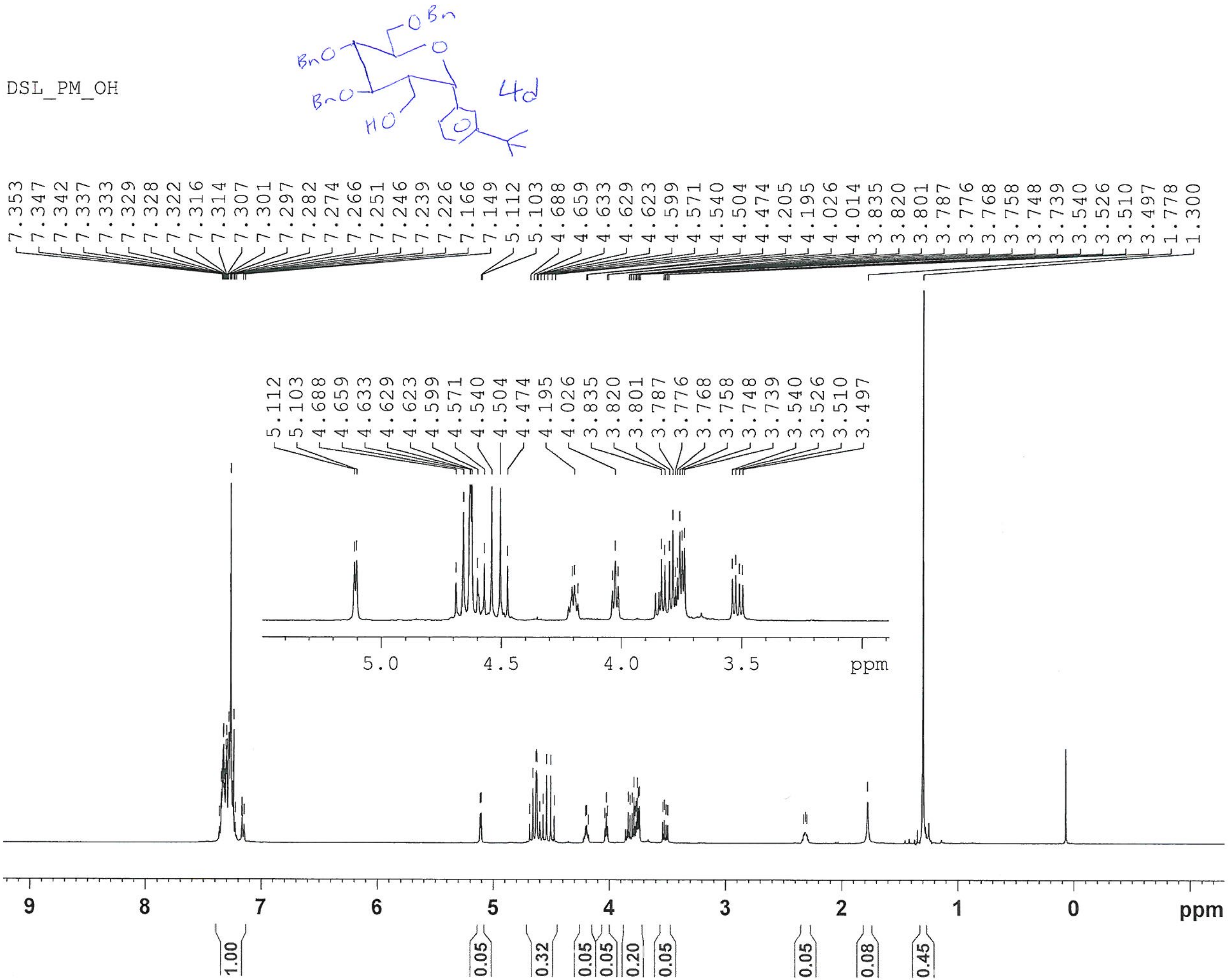
DSL_PM_OH
BnO
BnO
HO
OBn
O
4d
7.353
7.347
7.342
7.337
7.333
7.329
7.328
7.322
7.316
7.314
7.307
7.301
7.297
7.282
7.274
7.266
7.251
7.246
7.239
7.226
7.166
7.149
5.112
5.103
4.688
4.659
4.633
4.629
4.623
4.599
4.571
4.540
4.504
4.474
4.205
4.195
4.026
4.014
3.835
3.820
3.801
3.787
3.776
3.768
3.758
3.748
3.739
3.540
3.526
3.510
3.497
1.778
1.300
5.112
5.103
4.688
4.659
4.633
4.629
4.623
4.599
4.571
4.540
4.504
4.474
4.195
4.026
3.835
3.820
3.801
3.787
3.776
3.768
3.758
3.748
3.739
3.540
3.526
3.510
3.497
5.0
4.5
4.0
3.5
ppm
9
8
7
6
5
4
3
2
1
0
ppm
1.00
0.05
0.32
0.05
0.05
0.20
0.05
0.05
0.08
0.45

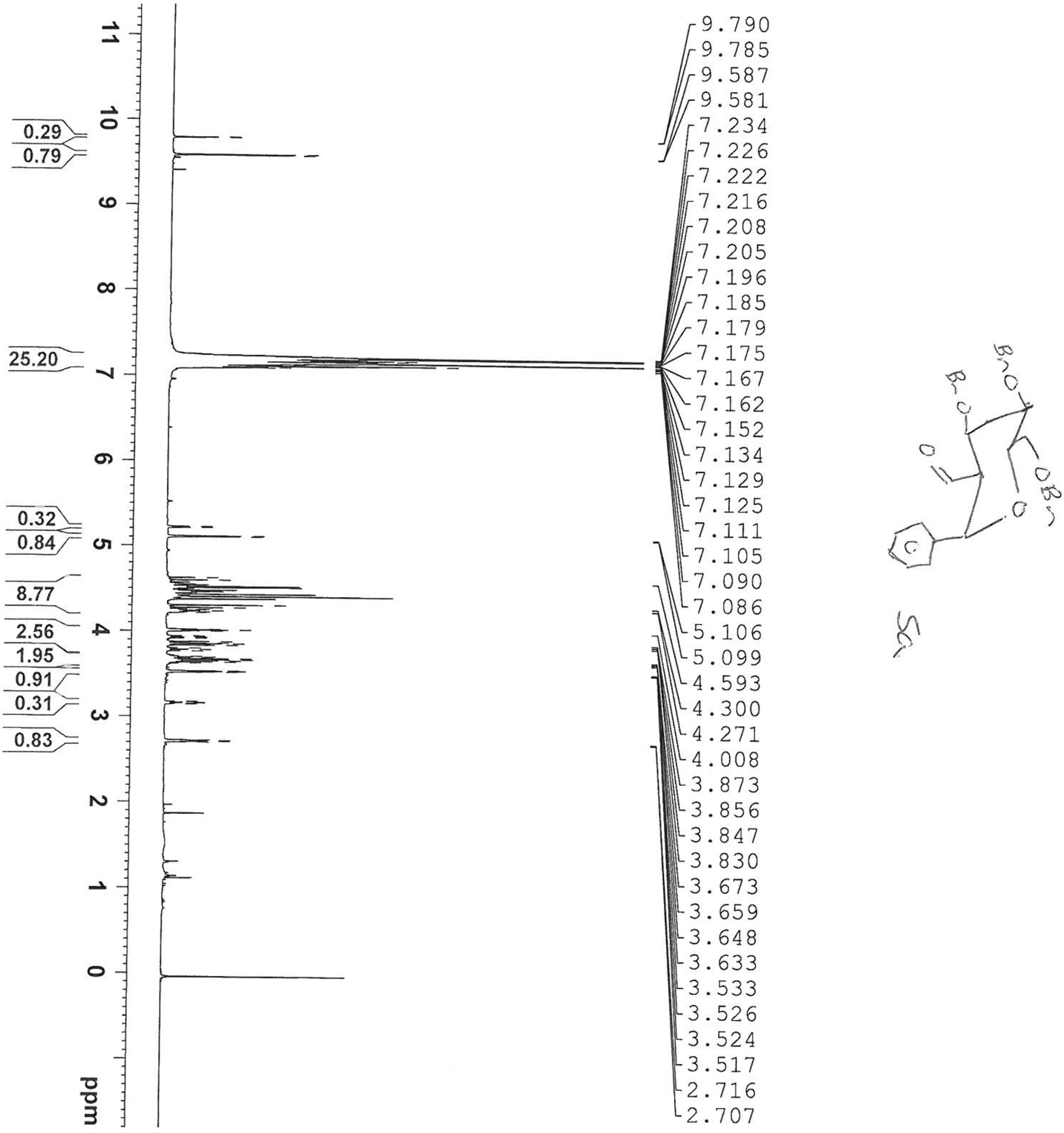
0.29
0.79
25.20
0.32
0.84
8.77
2.56
1.95
0.91
0.31
0.83
ppm
9.790
9.785
9.587
9.581
7.234
7.226
7.222
7.216
7.208
7.205
7.196
7.185
7.179
7.175
7.167
7.162
7.152
7.134
7.129
7.125
7.111
7.105
7.090
7.086
5.106
5.099
4.593
4.300
4.271
4.008
3.873
3.856
3.847
3.830
3.673
3.659
3.648
3.633
3.533
3.526
3.524
3.517
2.716
2.707

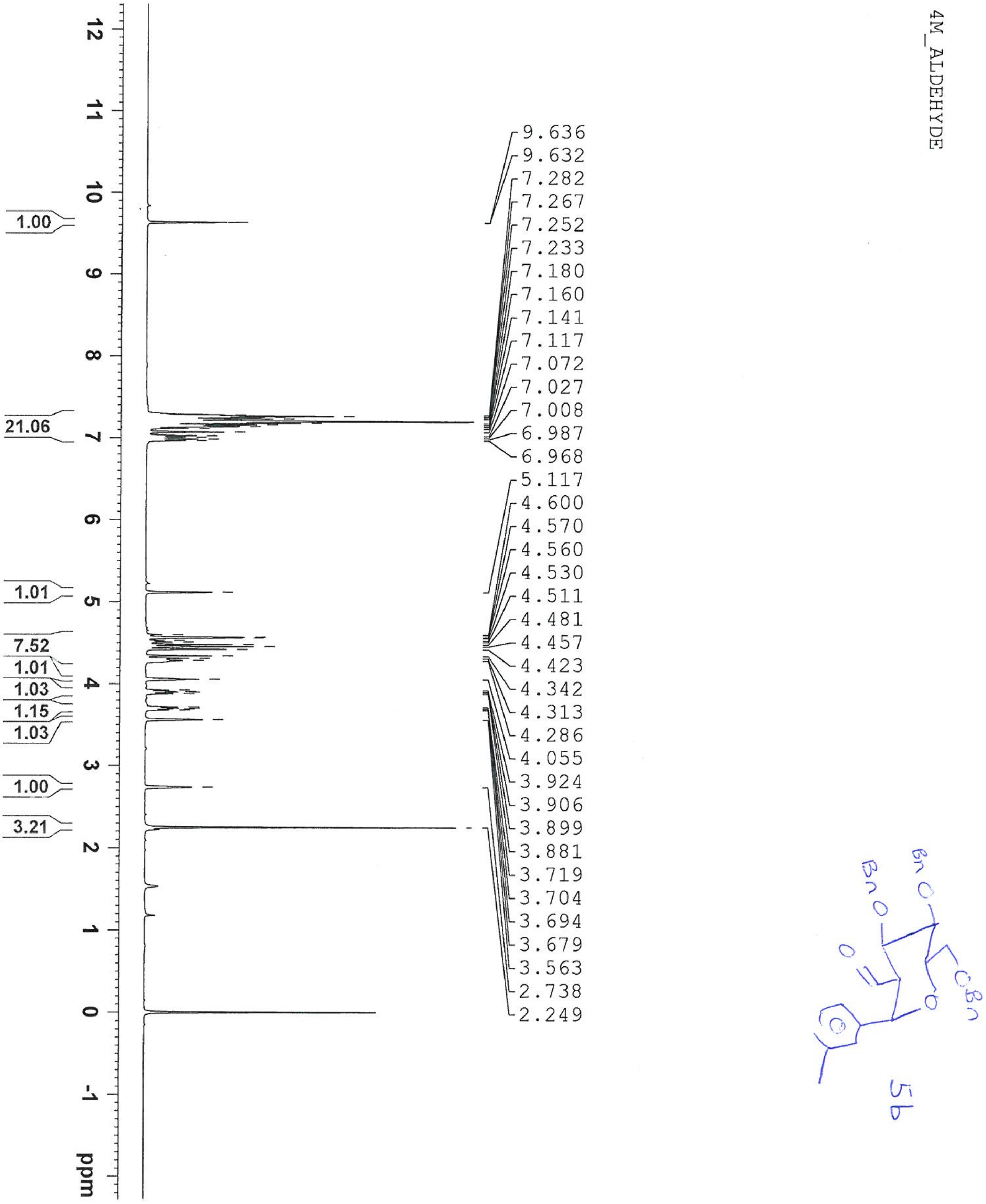
4M_ALDEHYDE
9.636
9.632
7.282
7.267
7.252
7.233
7.180
7.160
7.141
7.117
7.072
7.027
7.008
6.987
6.968
5.117
4.600
4.570
4.560
4.530
4.511
4.481
4.457
4.423
4.342
4.313
4.286
4.055
3.924
3.906
3.899
3.881
3.719
3.704
3.694
3.679
3.563
2.738
2.249
1.00
21.06
1.01
7.52
1.01
1.03
1.15
1.03
1.00
3.21
12
11
10
9
8
7
6
5
4
3
2
1
0
-1
ppm
BnO
BnO
OBn
5b

methoxy_al_rn

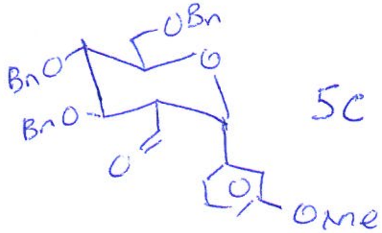

5c

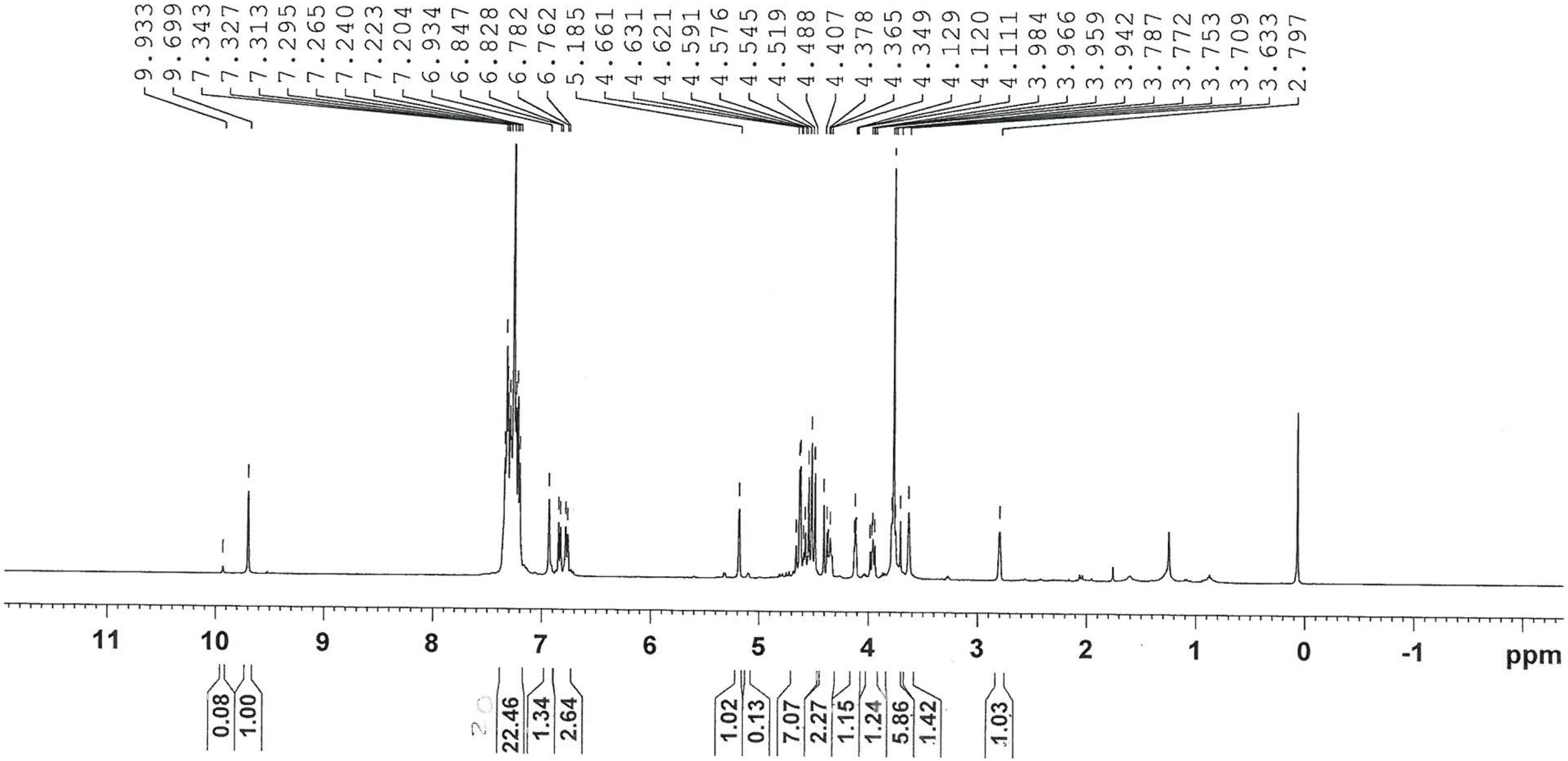

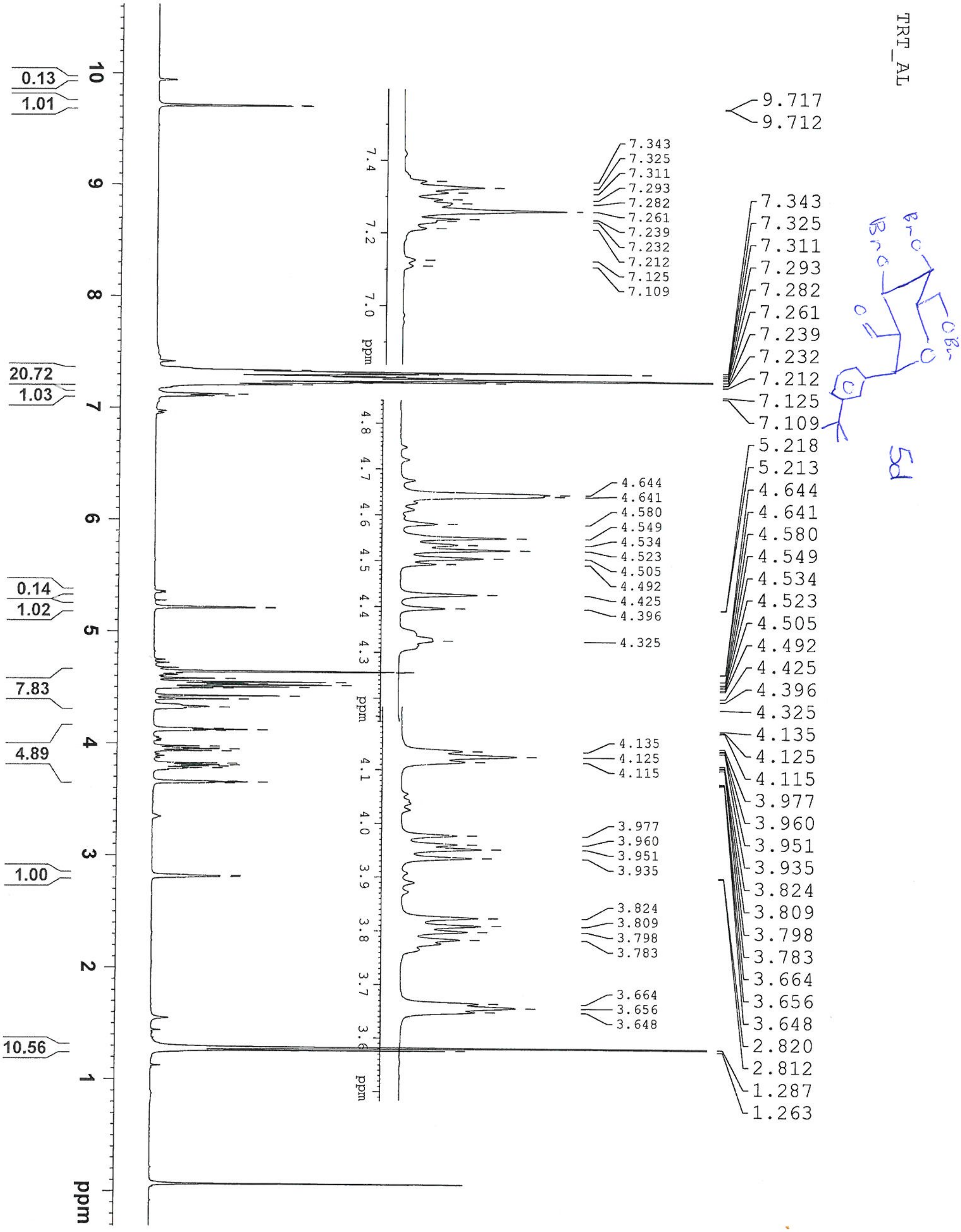

TRT_AL
9.717
9.712
7.343
7.325
7.311
7.293
7.282
7.261
7.239
7.232
7.212
7.125
7.109
5.218
5.213
4.644
4.641
4.580
4.549
4.534
4.523
4.505
4.492
4.425
4.396
4.325
4.135
4.125
4.115
3.977
3.960
3.951
3.935
3.824
3.809
3.798
3.783
3.664
3.656
3.648
2.820
2.812
1.287
1.263
5d
0.13
1.01
20.72
1.03
0.14
1.02
7.83
4.89
1.00
10.56
ppm

THIOPHENOL_FERRIER

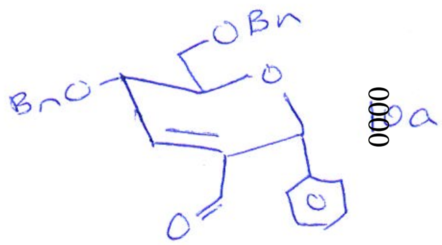

0000

9a

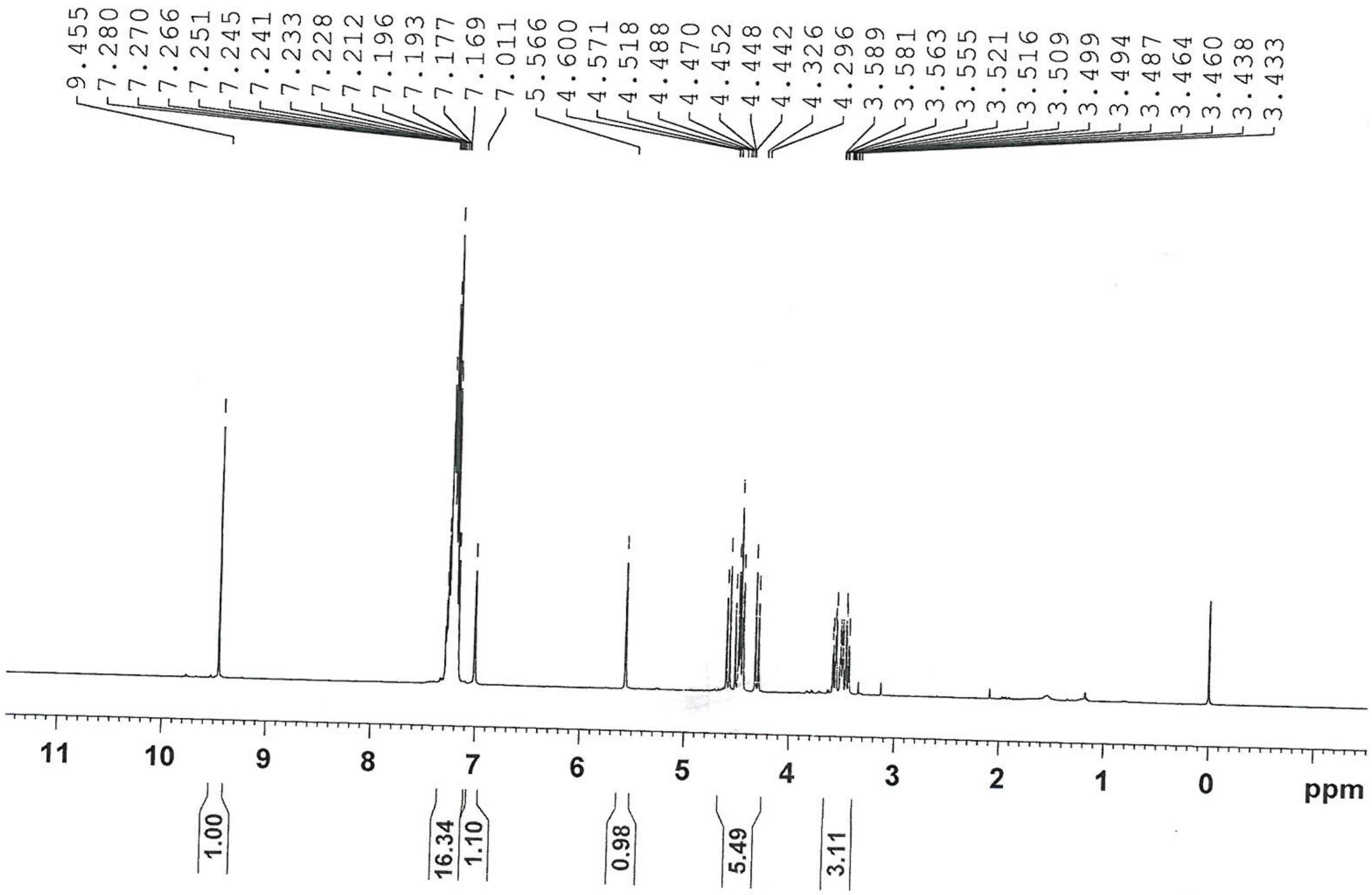

NAME	THIOPHENOL_FERRRIER
EXPNO	10
PROCNO	1
Date_	20140703
Time	18.09
INSTRUM	spect
PROBHD	5 mm PABBO BB-
PULPROG	zg30
TD	65536
SOLVENT	CDCl3
NS	32
DS	2
SWH	8223.685 Hz
FIDRES	0.125483 Hz
AQ	3.9846387 sec
RG	50.8
DW	60.800 usec
DE	6.50 usec
TE	286.8 K
D1	1.00000000 sec
TD0	1

======== CHANNEL f1 ========

NUC1	1H
P1	9.30 usec
PL1	-3.50 dB
SFO1	400.1724712 MHz
SI	32768
SF	400.1700446 MHz
WDW	EM
SSB	0
LB	0.30 Hz
GB	0
PC	1.00

4M_FERRIER

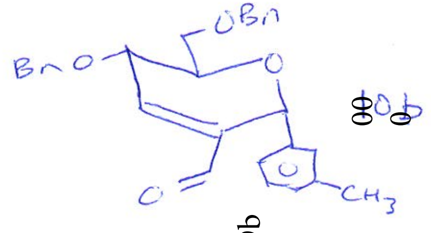

9b

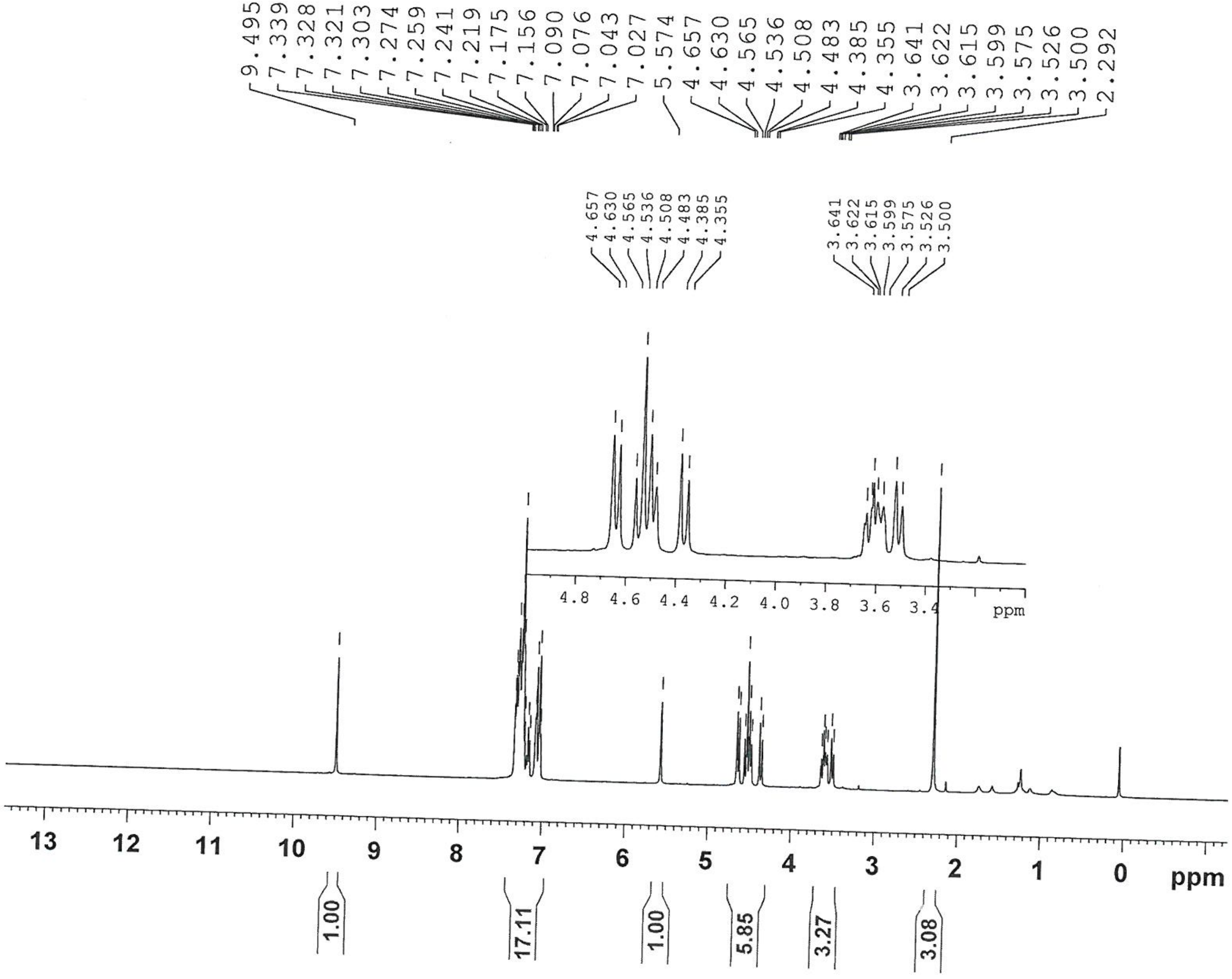

TRT_FERRIER

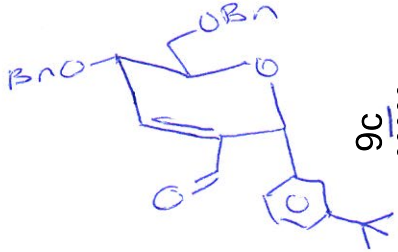

9c

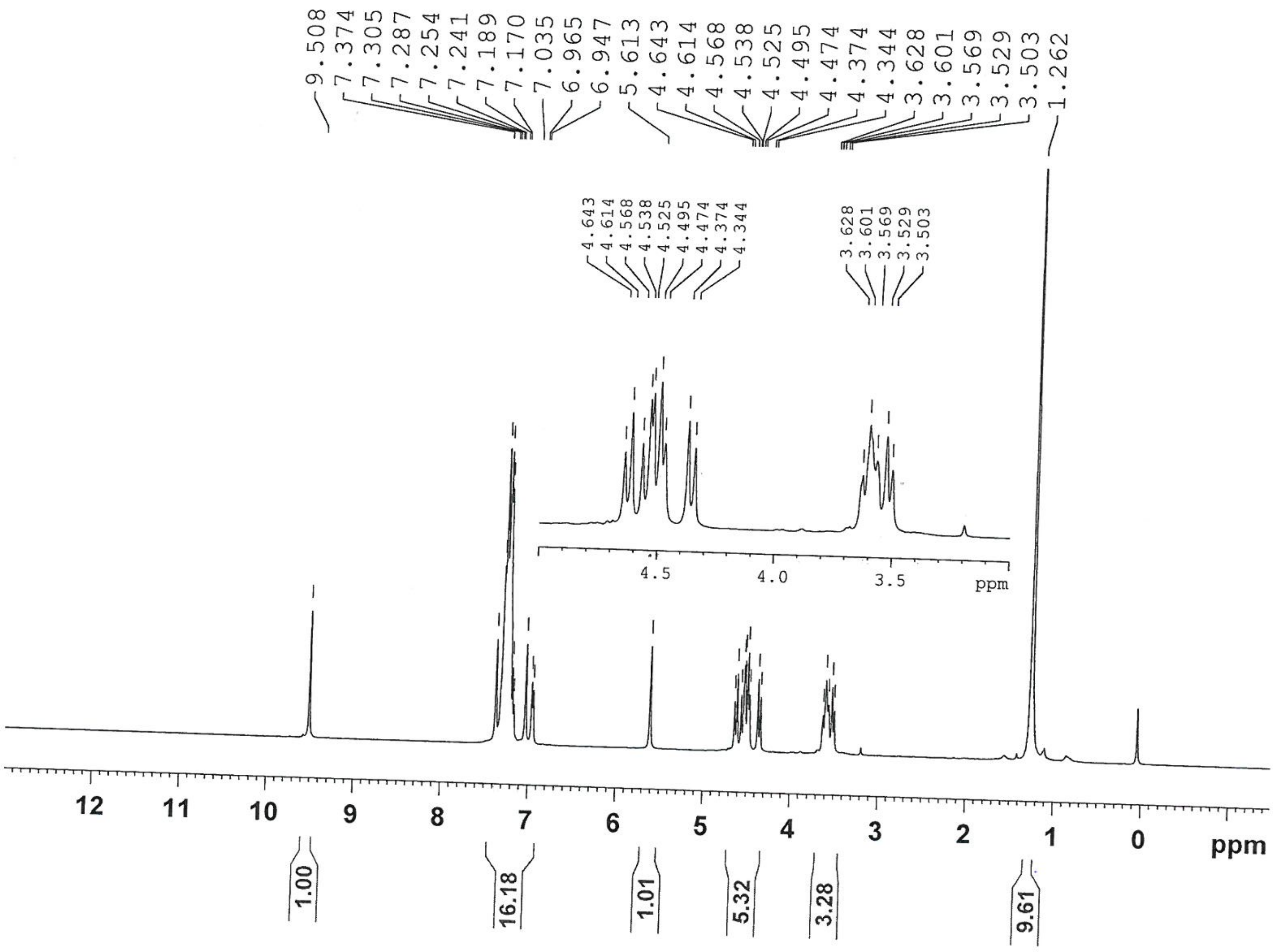

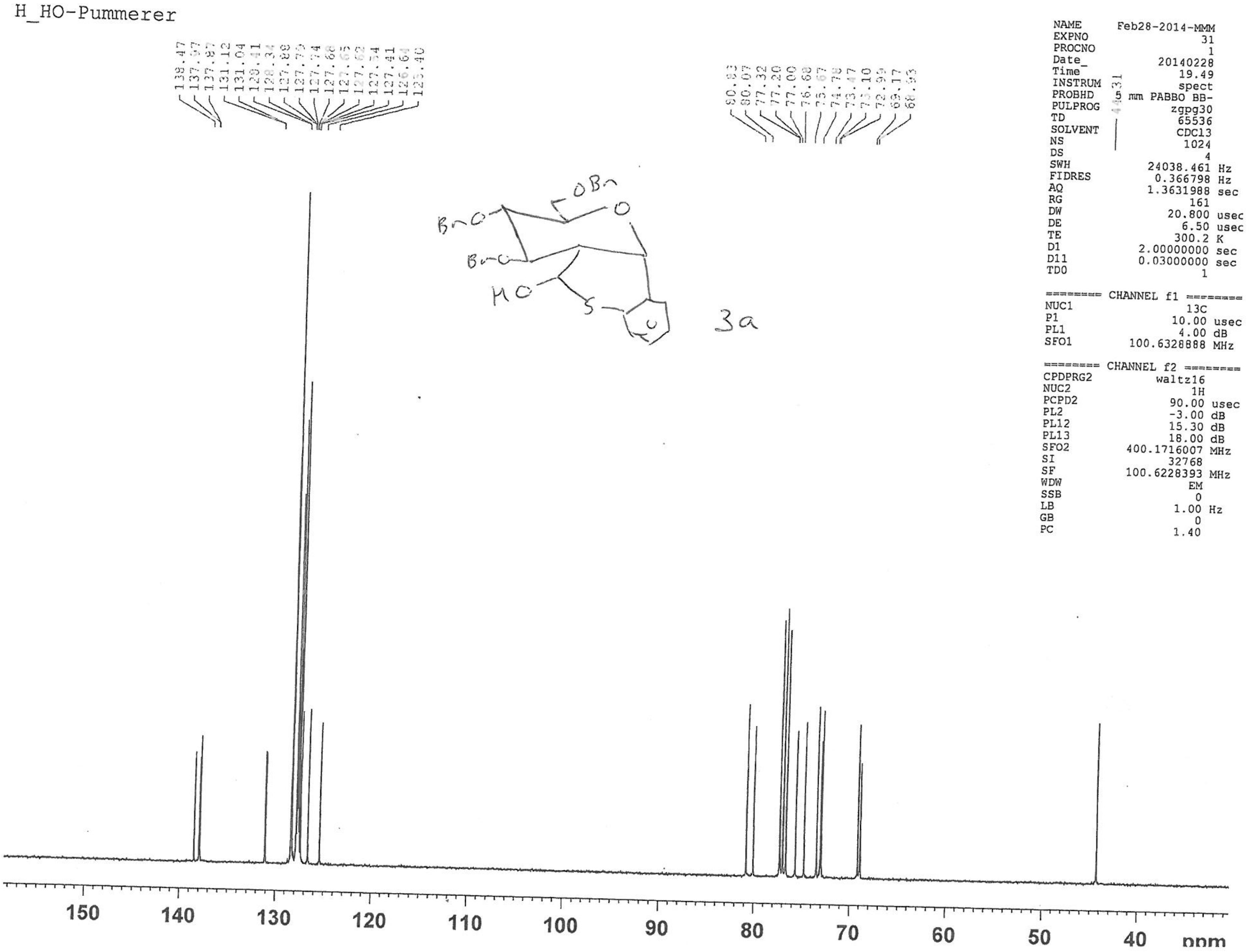
H_HO-Pummerer
138.47
137.97
137.87
131.12
131.04
128.41
128.34
127.88
127.79
127.74
127.68
127.65
127.62
127.54
127.41
126.64
125.40
80.83
80.07
77.32
77.20
77.00
76.68
75.67
74.78
73.47
73.10
72.99
69.17
68.93
44.31
NAME Feb28-2014-MMM
EXPNO 31
PROCNO 1
Date_ 20140228
Time 19.49
INSTRUM spect
PROBHD 5 mm PABBO BB-
PULPROG zgpg30
TD 65536
SOLVENT CDCl3
NS 1024
DS 4
SWH 24038.461 Hz
FIDRES 0.366798 Hz
AQ 1.3631988 sec
RG 161
DW 20.800 usec
DE 6.50 usec
TE 300.2 K
D1 2.00000000 sec
D11 0.03000000 sec
TD0 1
======== CHANNEL f1 ========
NUC1 13C
P1 10.00 usec
PL1 4.00 dB
SFO1 100.6328888 MHz
======== CHANNEL f2 ========
CPDPRG2 waltz16
NUC2 1H
PCPD2 90.00 usec
PL2 -3.00 dB
PL12 15.30 dB
PL13 18.00 dB
SFO2 400.1716007 MHz
SI 32768
SF 100.6228393 MHz
WDW EM
SSB 0
LB 1.00 Hz
GB 0
PC 1.40
OBn
BnO
BnO
HO
S
O
3a
150
140
130
120
110
100
90
80
70
60
50
40
ppm

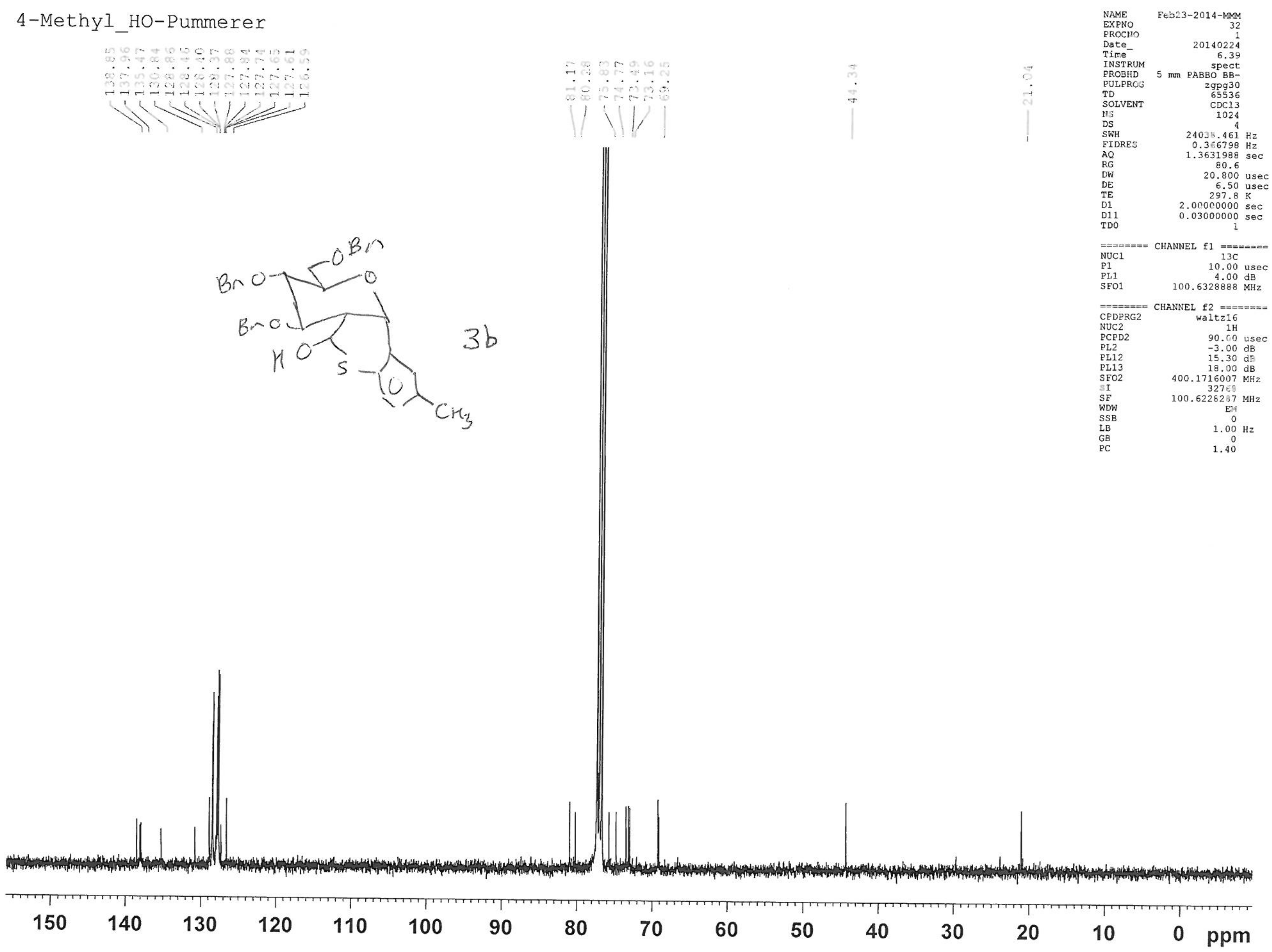
4-Methyl_HO-Pummerer
138.85
137.96
135.47
130.84
128.86
128.46
128.40
128.37
127.88
127.84
127.74
127.65
127.61
126.59
81.17
80.28
75.83
74.77
73.49
73.16
69.25
44.34
21.04
3b
NAME Feb23-2014-MMM
EXPNO 32
PROCNO 1
Date_ 20140224
Time 6.39
INSTRUM spect
PROBHD 5 mm PABBO BB-
PULPROG zgpg30
TD 65536
SOLVENT CDCl3
NS 1024
DS 4
SWH 24038.461 Hz
FIDRES 0.366798 Hz
AQ 1.3631988 sec
RG 80.6
DW 20.800 usec
DE 6.50 usec
TE 297.8 K
D1 2.00000000 sec
D11 0.03000000 sec
TD0 1
======== CHANNEL f1 ========
NUC1 13C
P1 10.00 usec
PL1 4.00 dB
SFO1 100.6328888 MHz
======== CHANNEL f2 ========
CPDPRG2 waltz16
NUC2 1H
PCPD2 90.00 usec
PL2 -3.00 dB
PL12 15.30 dB
PL13 18.00 dB
SFO2 400.1716007 MHz
SI 32768
SF 100.6226287 MHz
WDW EM
SSB 0
LB 1.00 Hz
GB 0
PC 1.40
150
140
130
120
110
100
90
80
70
60
50
40
30
20
10
0
ppm

4-MeO_HO-Pummerer

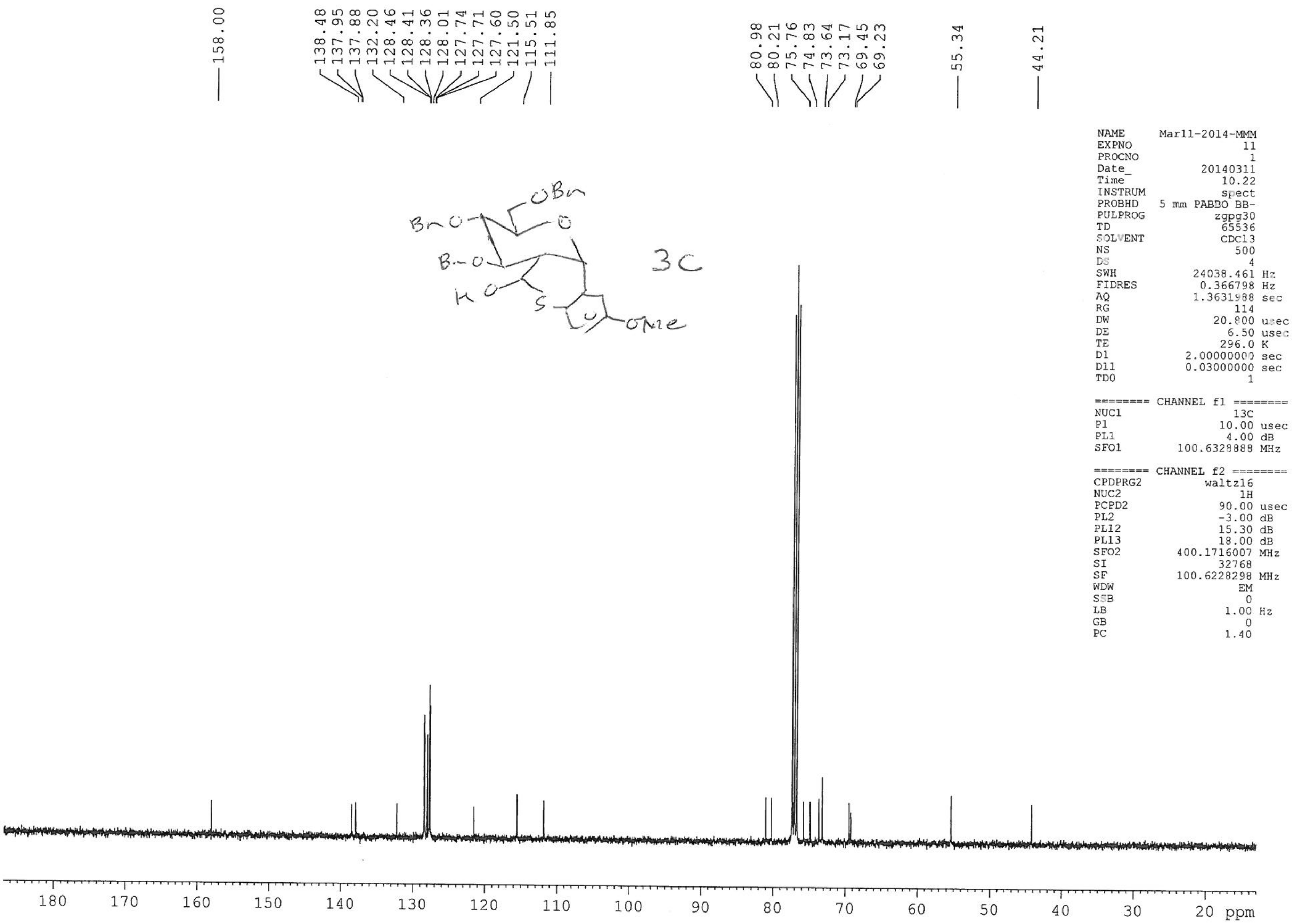

tert-Butyl_HO-Pum

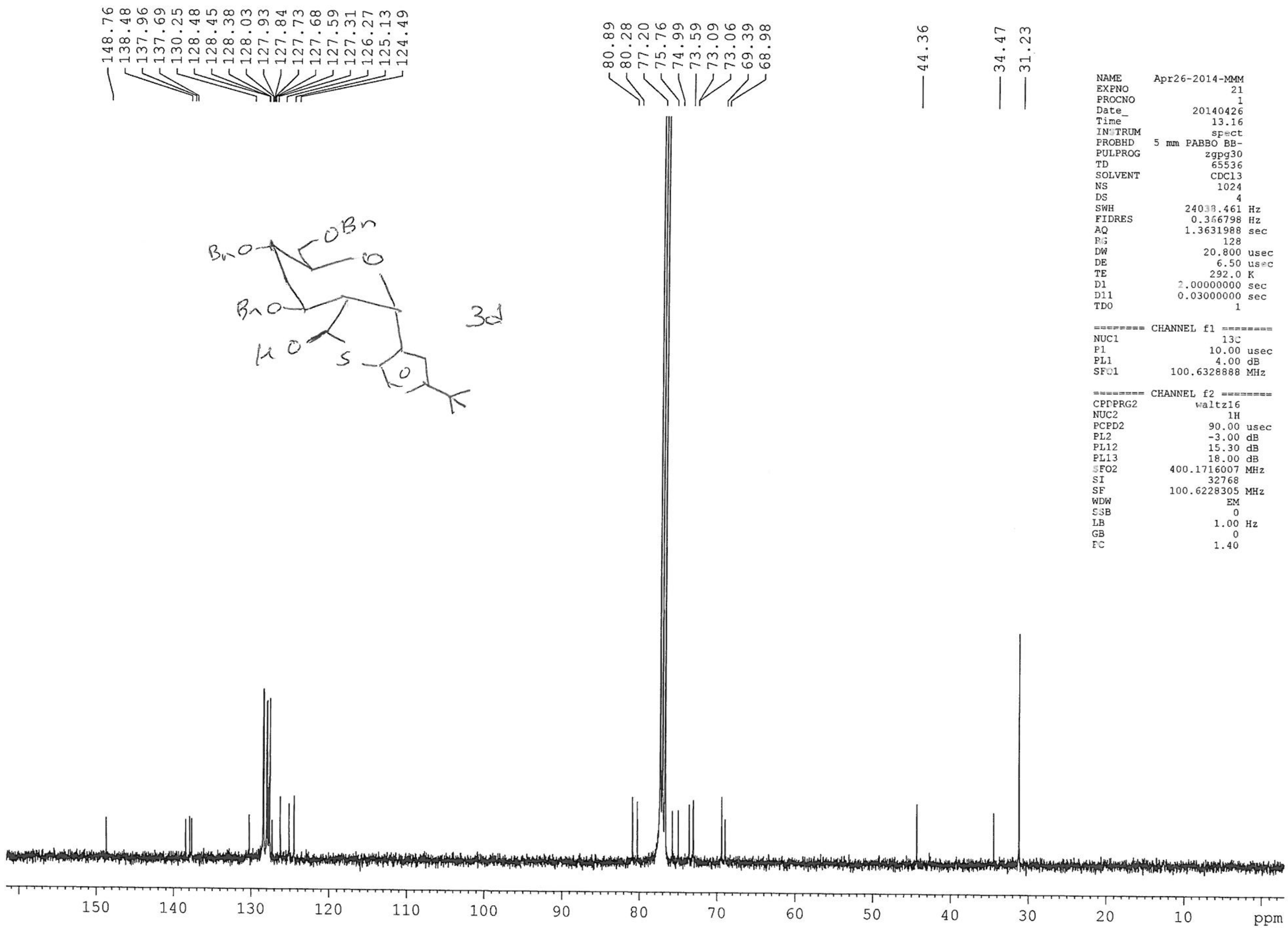

NAME	Apr26-2014-MMM
EXPNO	21
PROCNO	1
Date_	20140426
Time	13.16
INSTRUM	spect
PROBHD	5 mm PABBO BB-
PULPROG	zgpg30
TD	65536
SOLVENT	CDCl3
NS	1024
DS	4
SWH	24038.461 Hz
FIDRES	0.366798 Hz
AQ	1.3631988 sec
RG	128
DW	20.800 usec
DE	6.50 usec
TE	292.0 K
D1	2.00000000 sec
D11	0.03000000 sec
TD0	1
======== CHANNEL f1 ========	
NUC1	13C
P1	10.00 usec
PL1	4.00 dB
SFO1	100.6328888 MHz
======== CHANNEL f2 ========	
CPDPRG2	waltz16
NUC2	1H
PCPD2	90.00 usec
PL2	-3.00 dB
PL12	15.30 dB
PL13	18.00 dB
SFO2	400.1716007 MHz
SI	32768
SF	100.6228305 MHz
WDW	EM
SSB	0
LB	1.00 Hz
GB	0
PC	1.40

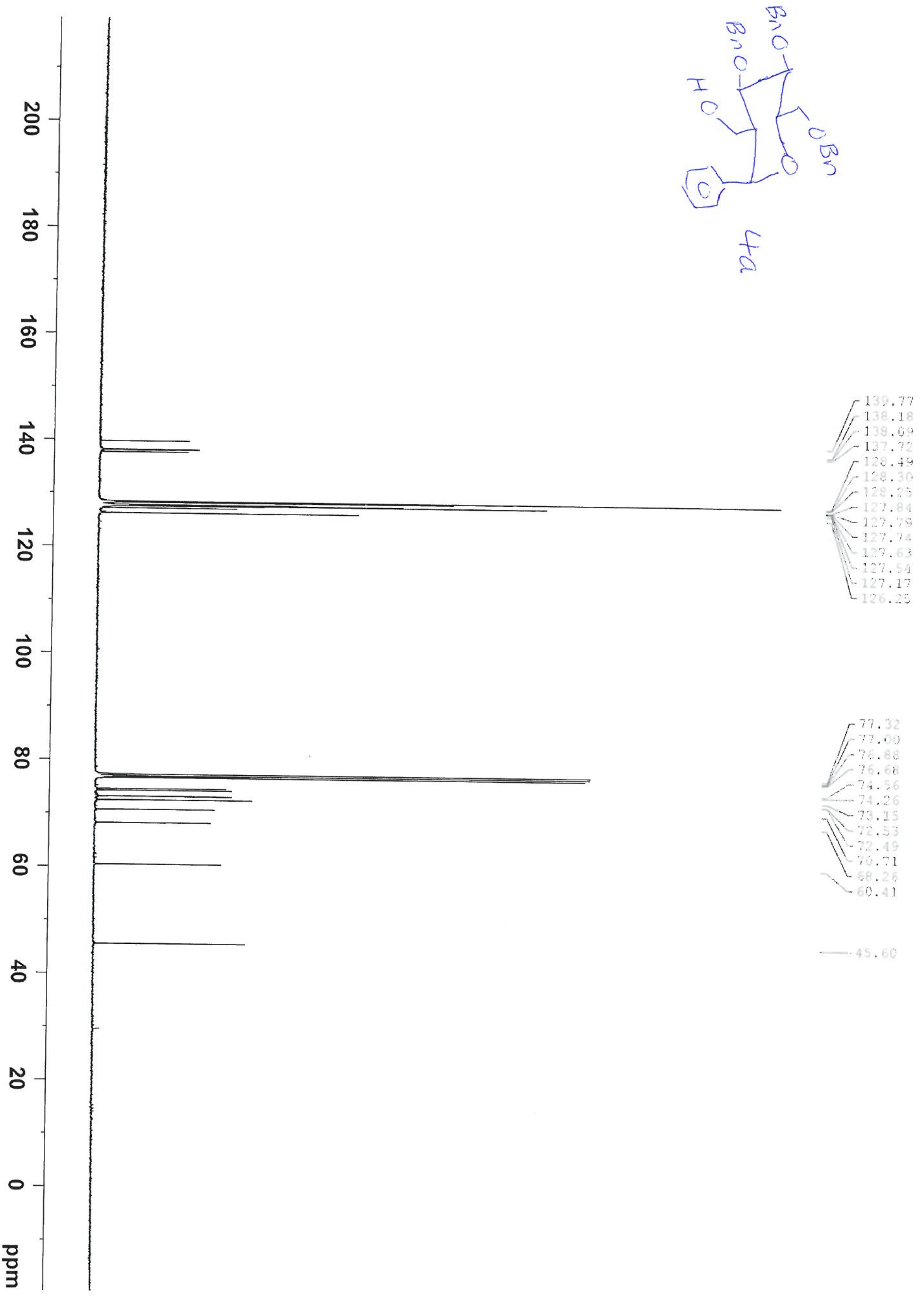
BnO
BnO
HO
OBn
O
4a
139.77
138.18
138.09
137.72
128.49
128.30
128.25
127.84
127.79
127.74
127.63
127.54
127.17
126.25
77.32
77.00
76.88
76.68
74.56
74.26
73.15
72.53
72.49
70.71
68.26
60.41
45.60
200
180
160
140
120
100
80
60
40
20
0
ppm

4M_OH

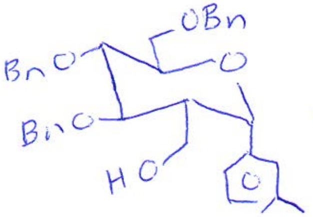

4b

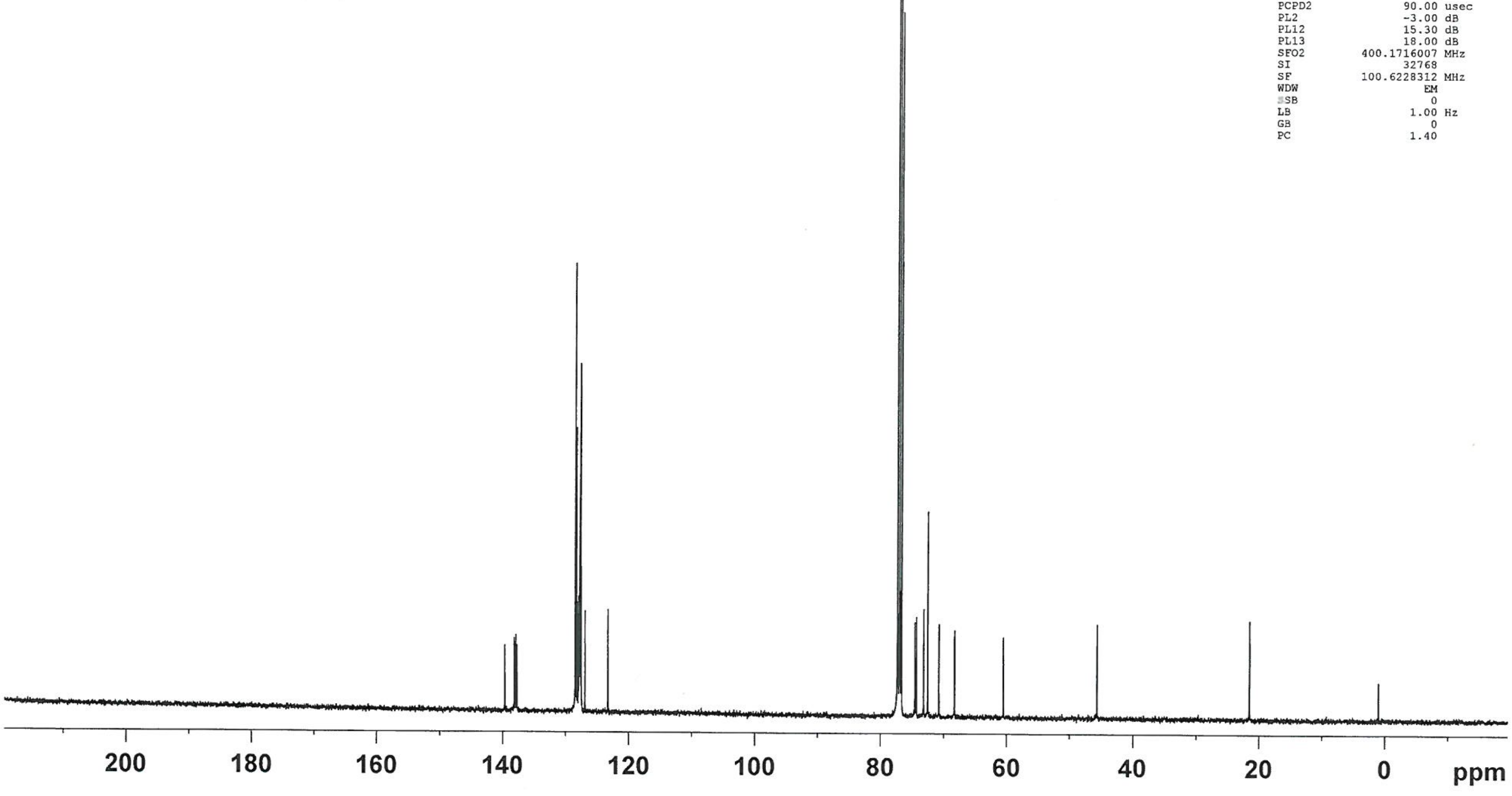

NAME	4M_OH
EXPNO	20
PROCNO	1
Date_	20140605
Time	2.20
INSTRUM	spect
PROBHD	5 mm PABBO BB-
PULPROG	zgpg30
TD	65536
SOLVENT	CDCl3
NS	2000
DS	4
SWH	24038.461 Hz
FIDRES	0.366798 Hz
AQ	1.3631988 sec
RG	144
DW	20.800 usec
DE	6.50 usec
TE	294.8 K
D1	2.00000000 sec
D11	0.03000000 sec
TD0	1
======== CHANNEL f1 ========	
NUC1	13C
P1	10.00 usec
PL1	4.00 dB
SFO1	100.6328888 MHz
======== CHANNEL f2 ========	
CPDPRG2	waltz16
NUC2	1H
PCPD2	90.00 usec
PL2	-3.00 dB
PL12	15.30 dB
PL13	18.00 dB
SFO2	400.1716007 MHz
SI	32768
SF	100.6228312 MHz
WDW	EM
SSB	0
LB	1.00 Hz
GB	0
PC	1.40

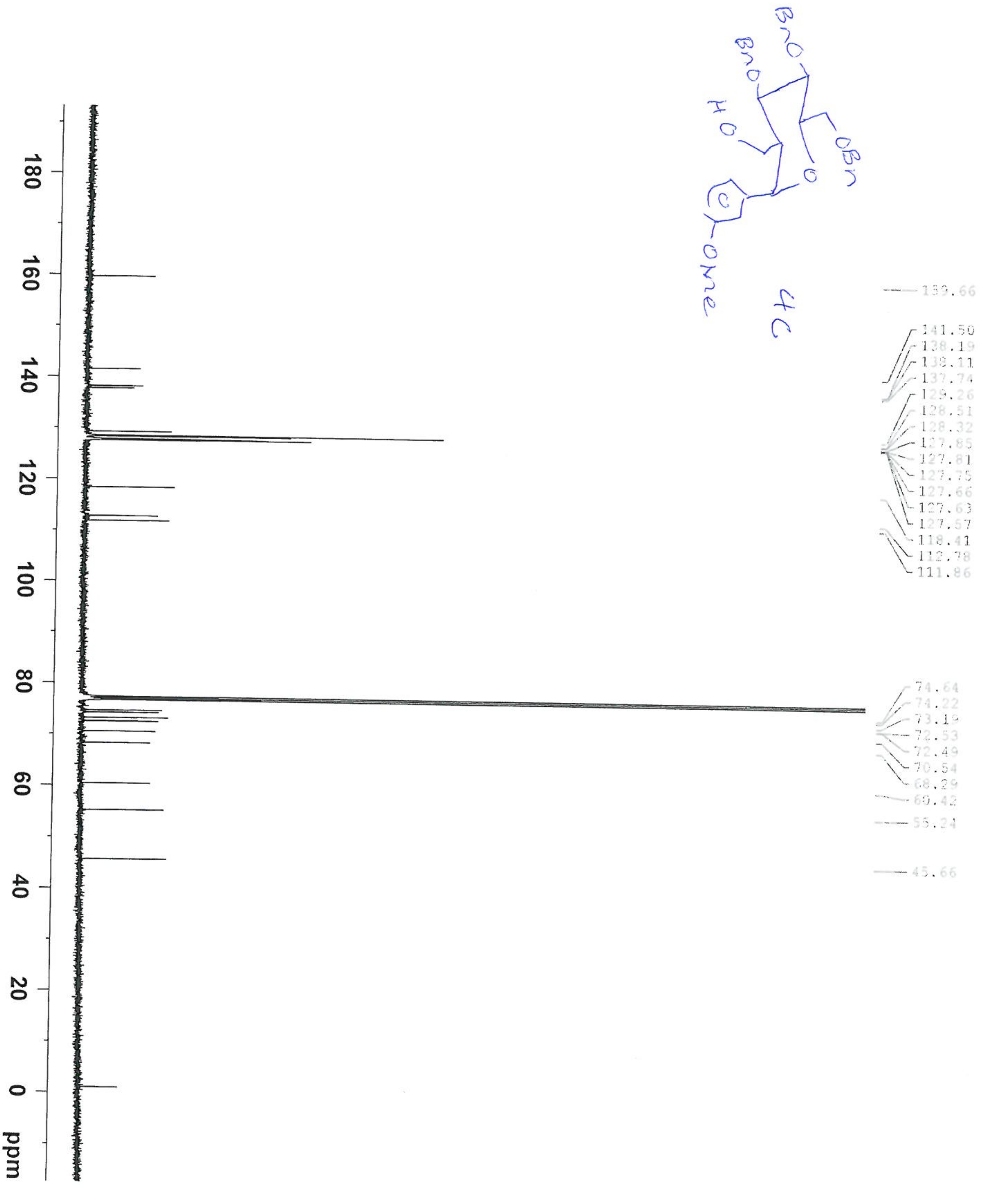
BnO
BnO
HO
OBn
O
OMe
4c
159.66
141.50
138.19
138.11
137.74
129.26
128.51
128.32
127.85
127.81
127.75
127.66
127.63
127.57
118.41
112.78
111.86
74.64
74.22
73.19
72.53
72.49
70.54
68.29
60.42
55.24
45.66
180
160
140
120
100
80
60
40
20
0
ppm

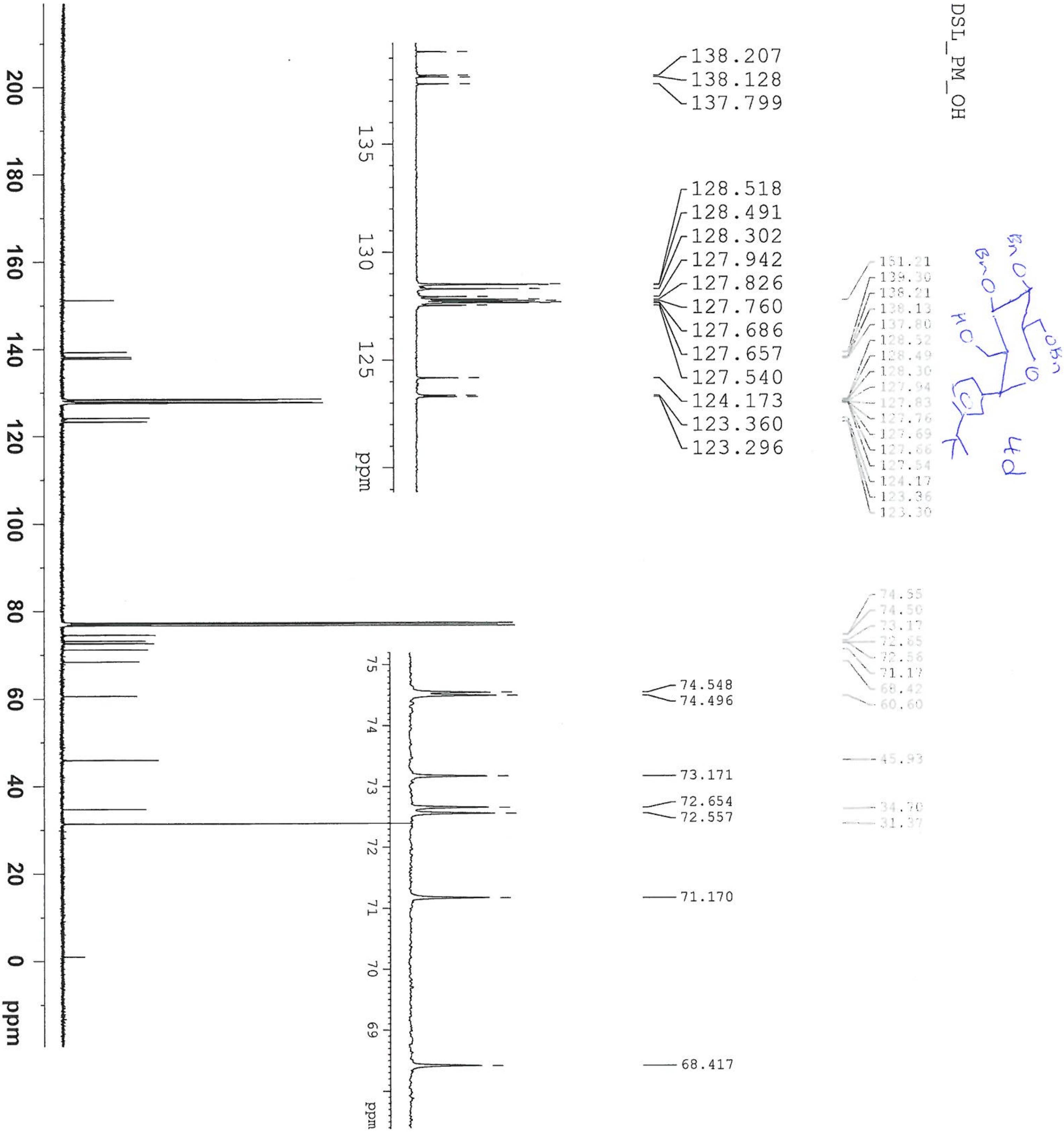
DSL_PM_OH
138.207
138.128
137.799
128.518
128.491
128.302
127.942
127.826
127.760
127.686
127.657
127.540
124.173
123.360
123.296
74.548
74.496
73.171
72.654
72.557
71.170
68.417
4d
ppm

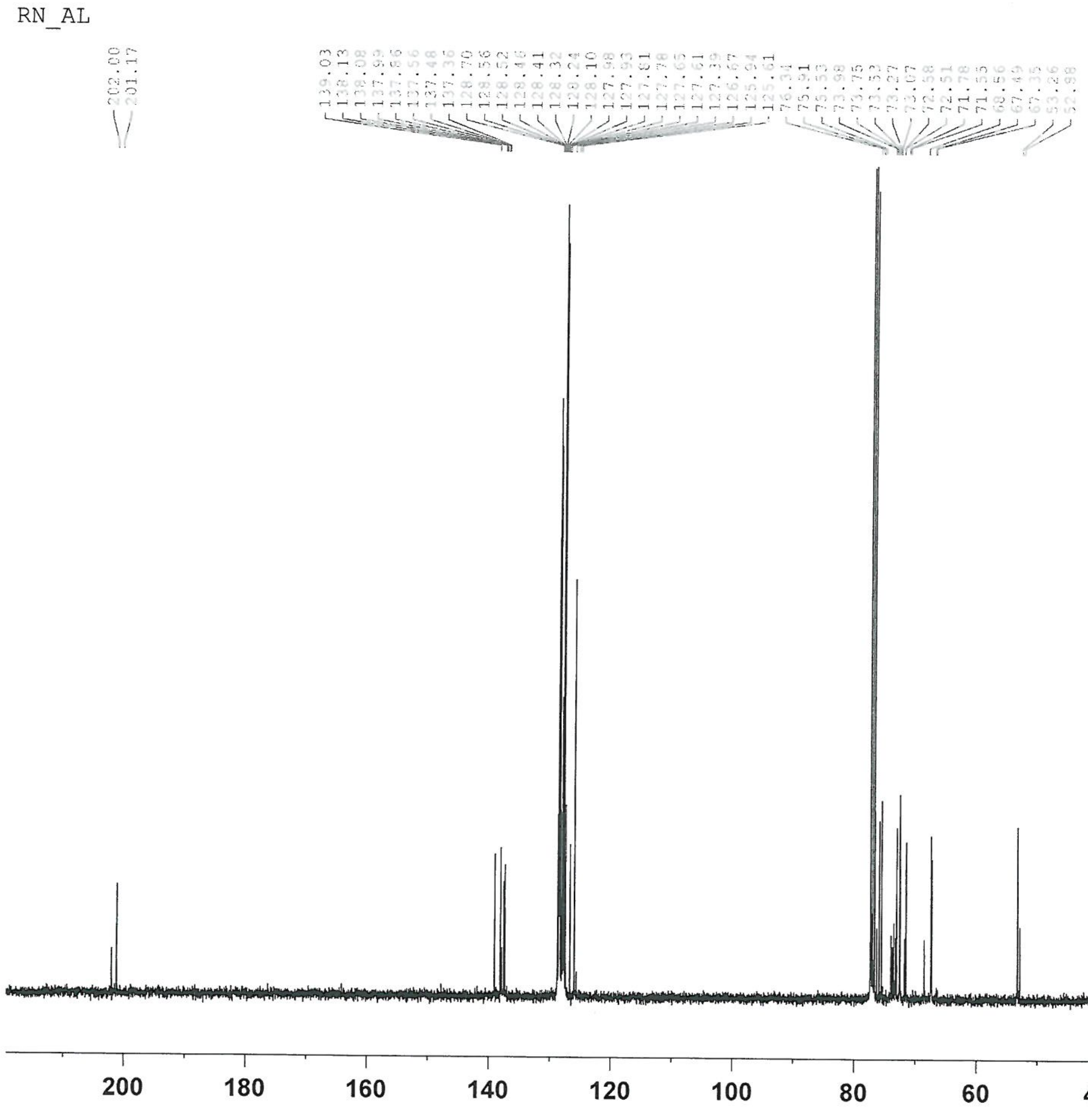
RN_AL
202.00
201.17
139.03
138.13
138.08
137.99
137.86
137.56
137.48
137.36
128.70
128.56
128.52
128.46
128.41
128.32
128.24
128.10
127.98
127.93
127.81
127.78
127.65
127.61
127.39
126.67
125.94
125.61
76.34
75.91
75.53
73.98
73.75
73.53
73.27
73.07
72.58
72.51
71.78
71.55
68.56
67.49
67.35
53.26
52.98
200
180
160
140
120
100
80
60
40
20
0
ppm

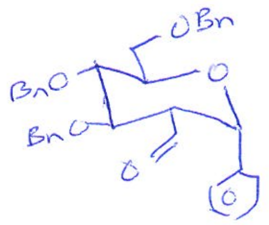
OBn
BnO
BnO
O
O

5a

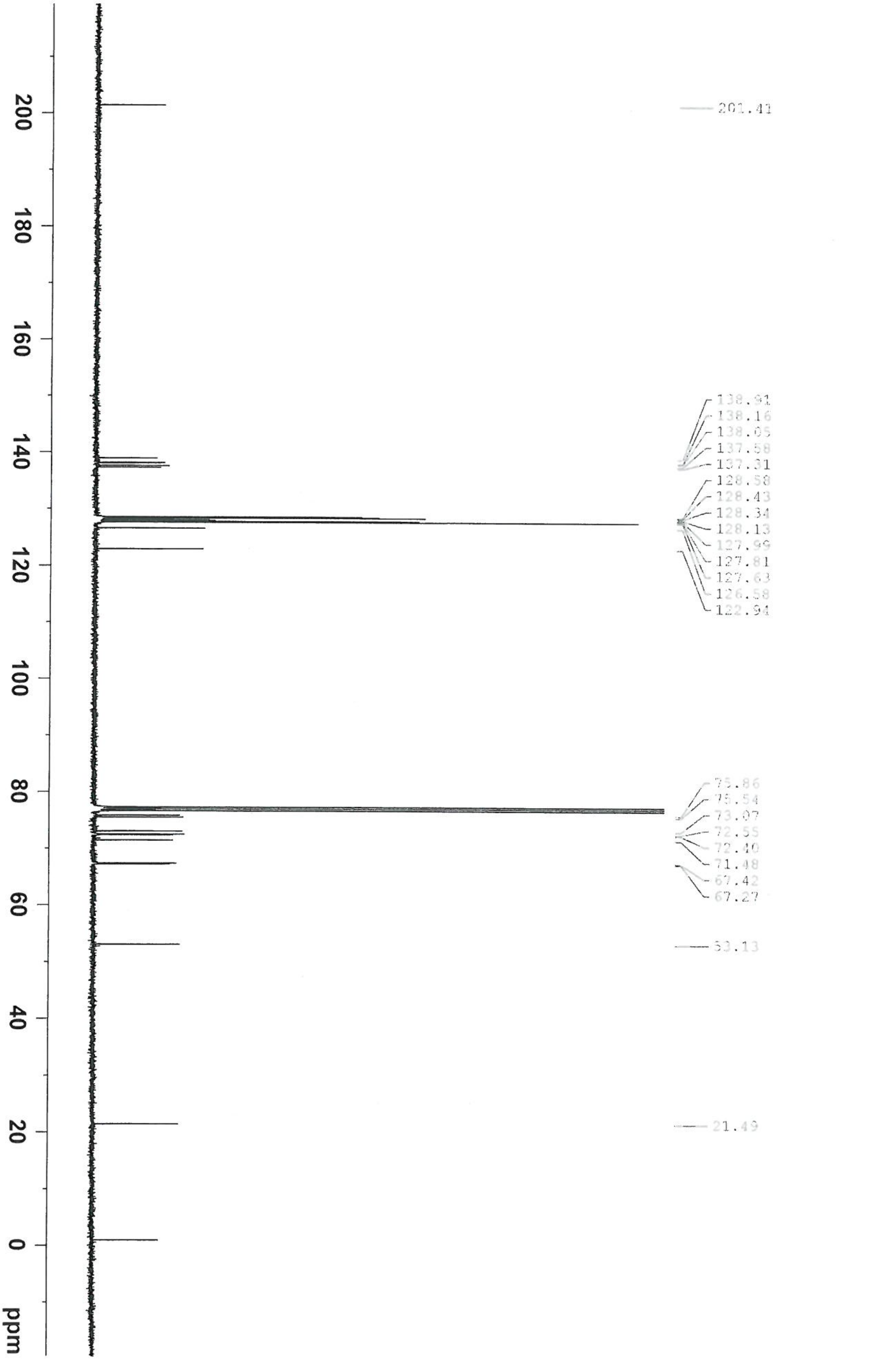
4M_ALDEHYDE
201.41
138.91
138.16
138.05
137.58
137.31
128.58
128.43
128.34
128.13
127.99
127.81
127.63
126.58
122.94
75.86
75.54
73.07
72.55
72.40
71.48
67.42
67.27
53.13
21.49
200
180
160
140
120
100
80
60
40
20
0
ppm

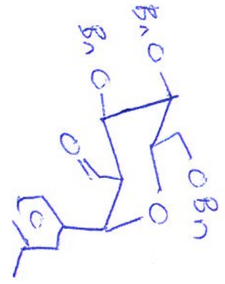
BnO
BnO
O
OBn
O

5b

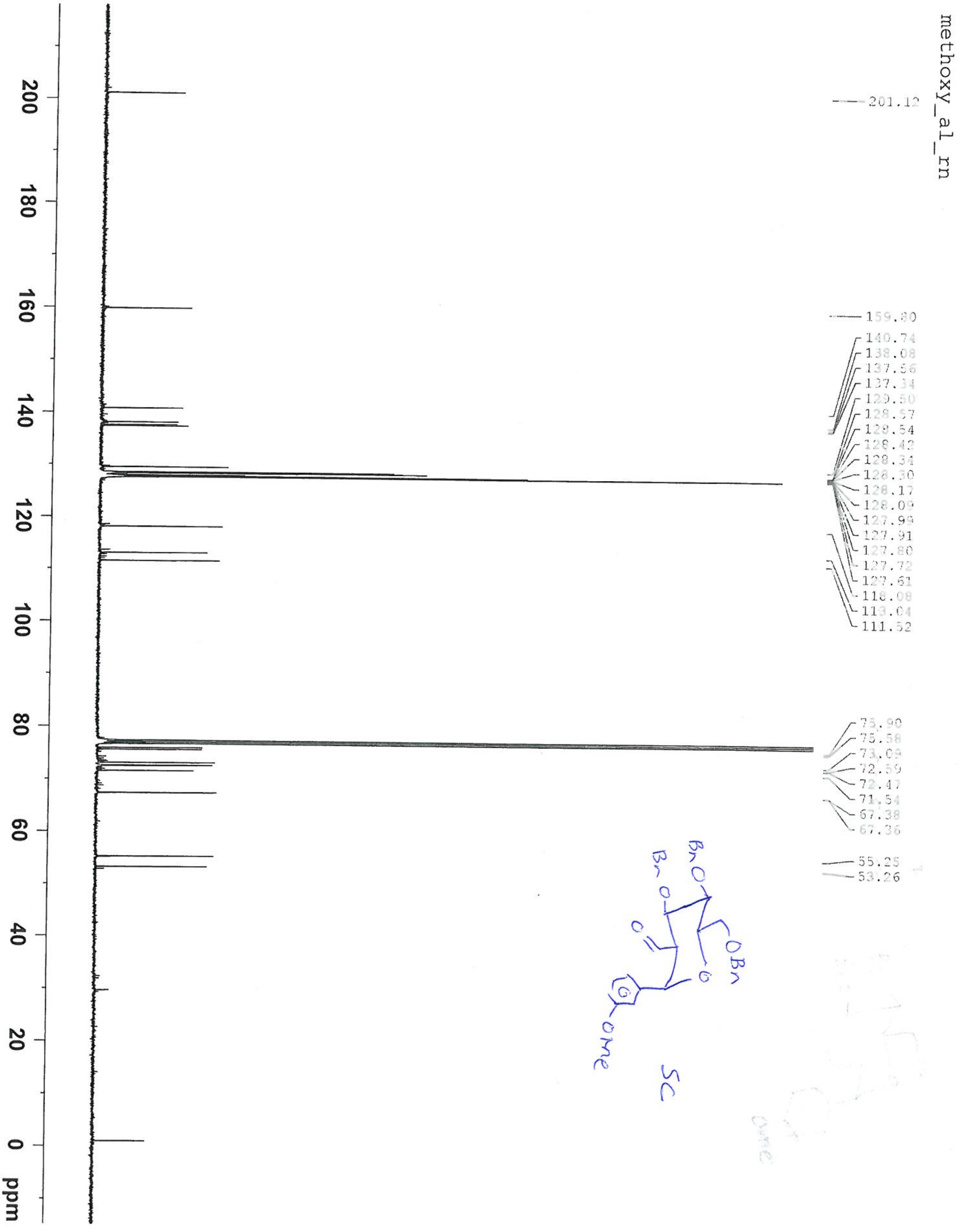
methoxy_al_rn
201.12
159.80
140.74
138.08
137.56
137.34
129.50
128.57
128.54
128.42
128.34
128.30
128.17
128.09
127.99
127.91
127.80
127.72
127.61
118.08
113.04
111.52
75.90
75.58
73.09
72.59
72.47
71.54
67.38
67.36
55.25
53.26
200
180
160
140
120
100
80
60
40
20
0
ppm
BnO
BnO
O=
OBn
O
OMe
5C

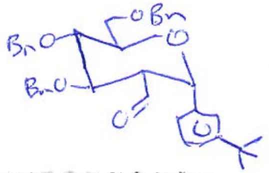

5d

TRT_AL

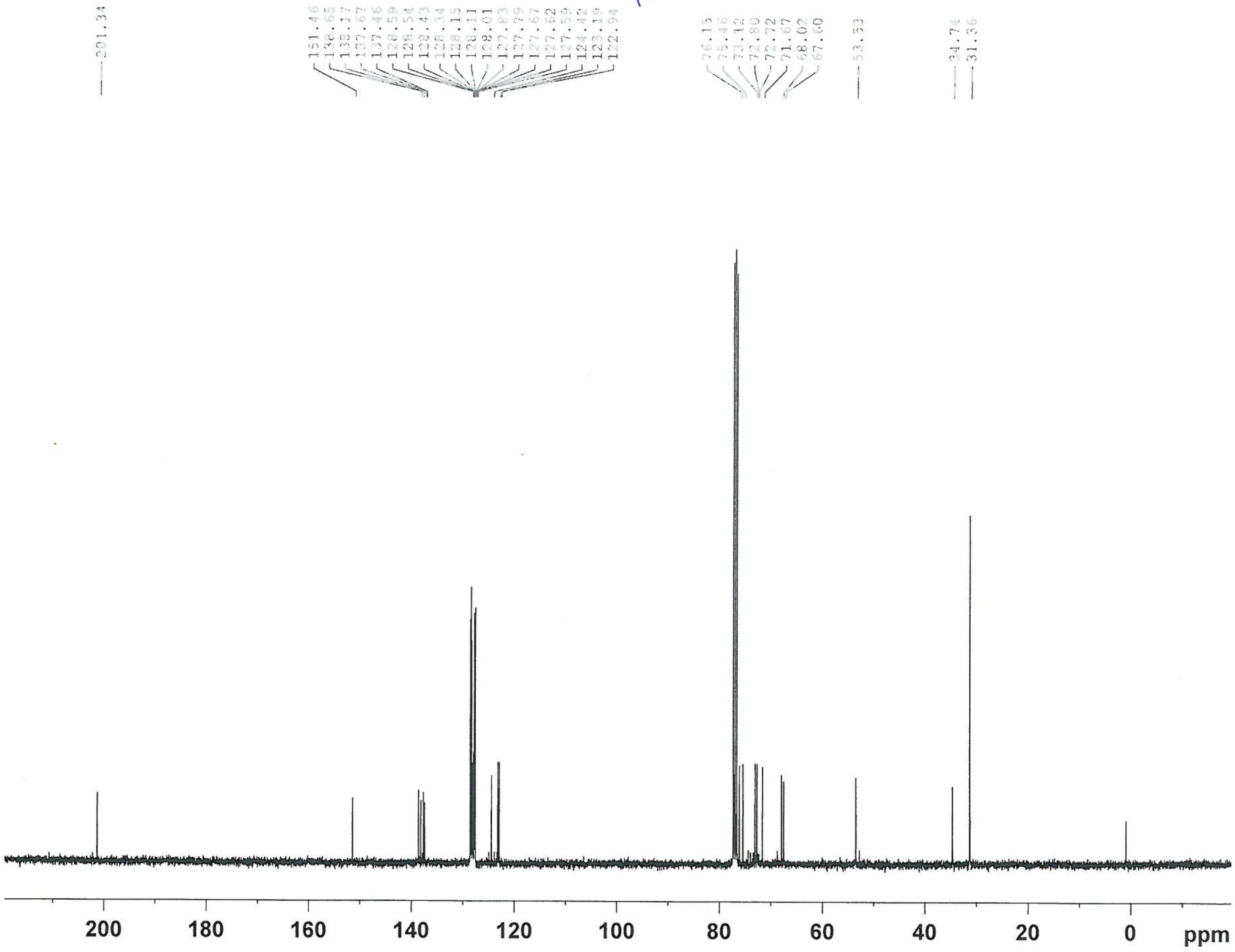
201.34
151.46
138.65
138.17
137.67
137.46
128.59
128.54
128.43
128.34
128.15
128.11
128.01
127.83
127.79
127.67
127.62
127.59
124.42
123.19
122.94
76.15
75.46
73.12
72.80
72.72
71.67
68.02
67.60
53.53
34.74
31.36
200
180
160
140
120
100
80
60
40
20
0
ppm

THIOPHENOL_FERRIER

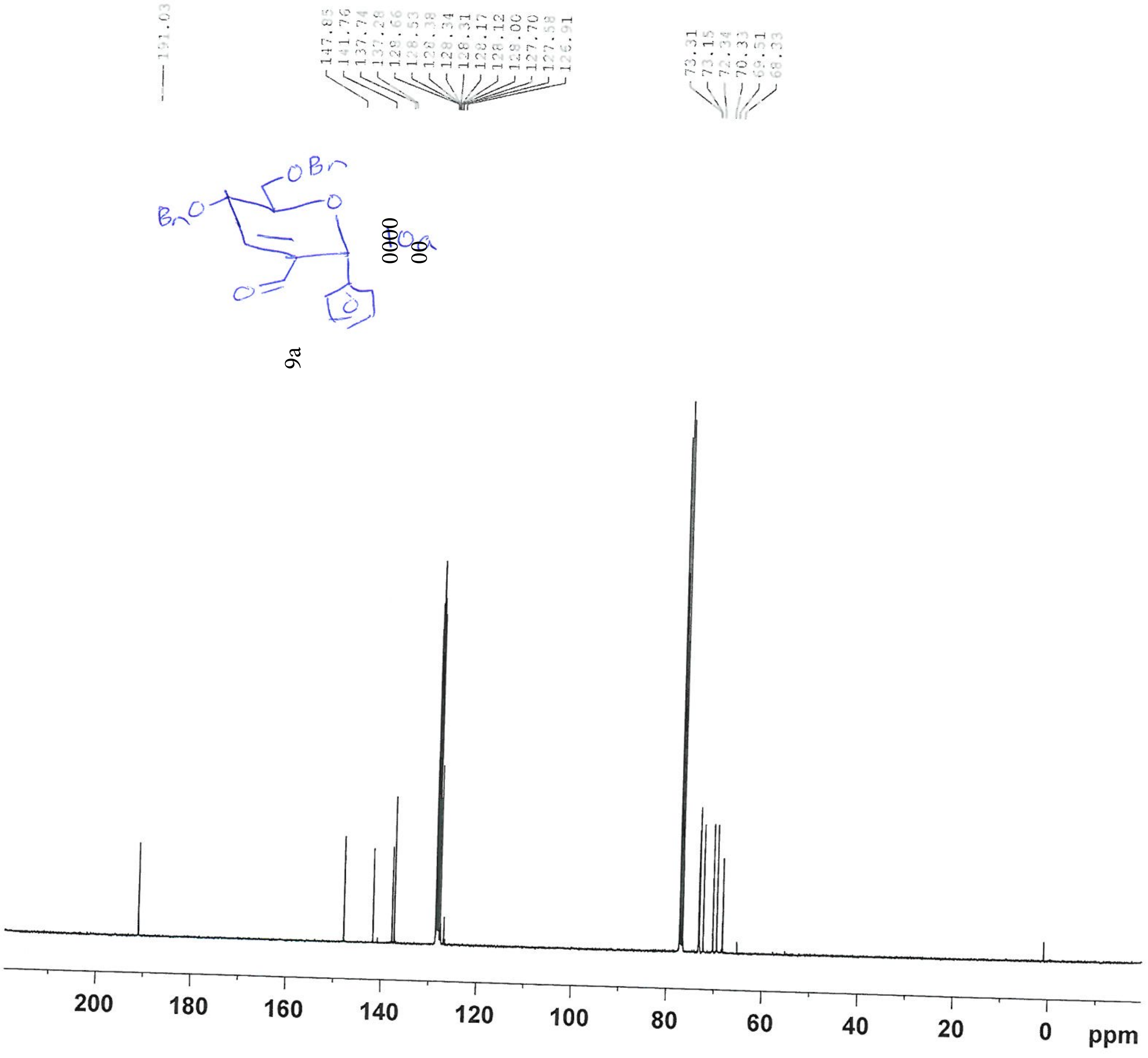

9a

```
NAME      THIOPHENOL_FERRRIER
EXPNO                  11
PROCNO                  1
Date_            20140703
Time                21.02
INSTRUM             spect
PROBHD     5 mm PABBO BB-
PULPROG            zgpg30
TD                  65536
SOLVENT             CDCl3
NS                   3000
DS                      4
SWH             24038.461 Hz
FIDRES           0.366798 Hz
AQ              1.3631988 sec
RG                    101
DW                 20.800 usec
DE                   6.50 usec
TE                  287.6 K
D1             2.00000000 sec
D11            0.03000000 sec
TD0                     1

======== CHANNEL f1 ========
NUC1                  13C
P1                  10.00 usec
PL1                  4.00 dB
SFO1          100.6328888 MHz

======== CHANNEL f2 ========
CPDPRG2           waltz16
NUC2                   1H
PCPD2               90.00 usec
PL2                 -3.00 dB
PL12                15.30 dB
PL13                18.00 dB
SFO2          400.1716007 MHz
SI                  32768
SF            100.6228370 MHz
WDW                    EM
SSB                     0
LB                   1.00 Hz
GB                      0
PC                   1.40
```

4M_FERRIER

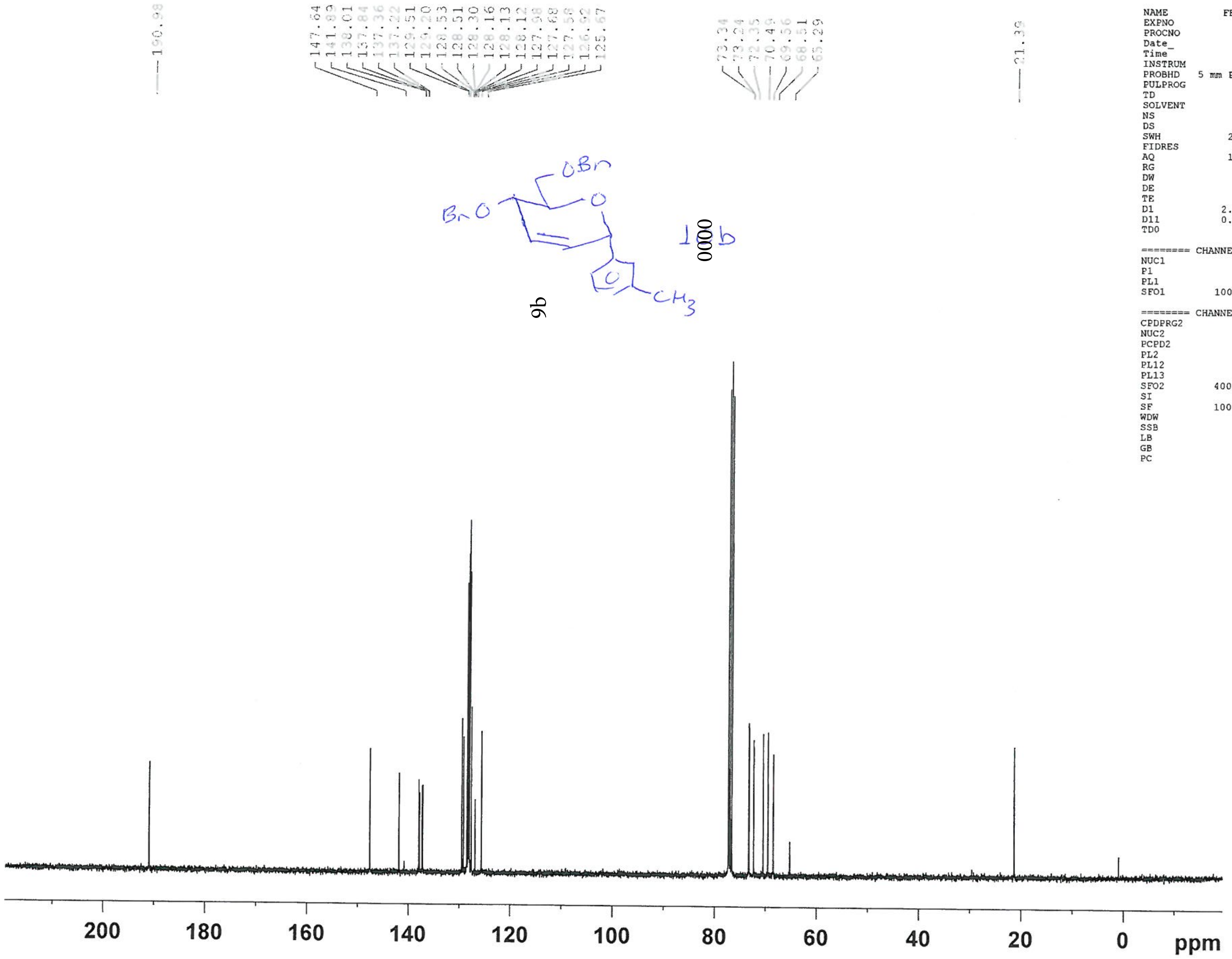

9b

0000

```
NAME         FERREIR_4M
EXPNO                11
PROCNO                1
Date_          20140626
Time              18.14
INSTRUM           spect
PROBHD   5 mm PABBO BB-
PULPROG         zgpg30
TD                65536
SOLVENT           CDCl3
NS                  500
DS                    4
SWH           24038.461 Hz
FIDRES         0.366798 Hz
AQ            1.3631988 sec
RG                  101
DW               20.800 usec
DE                 6.50 usec
TE                295.8 K
D1           2.00000000 sec
D11          0.03000000 sec
TD0                   1

======== CHANNEL f1 ========
NUC1                13C
P1                10.00 usec
PL1                4.00 dB
SFO1        100.6328898 MHz

======== CHANNEL f2 ========
CPDPRG2          waltz16
NUC2                  1H
PCPD2              90.00 usec
PL2                -3.00 dB
PL12               15.30 dB
PL13               18.00 dB
SFO2        400.1716007 MHz
SI                 32768
SF          100.6228341 MHz
WDW                   EM
SSB                    0
LB                  1.00 Hz
GB                     0
PC                  1.40
```

TRT_FERRIER

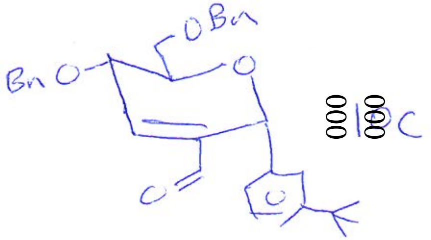

000
000

9c

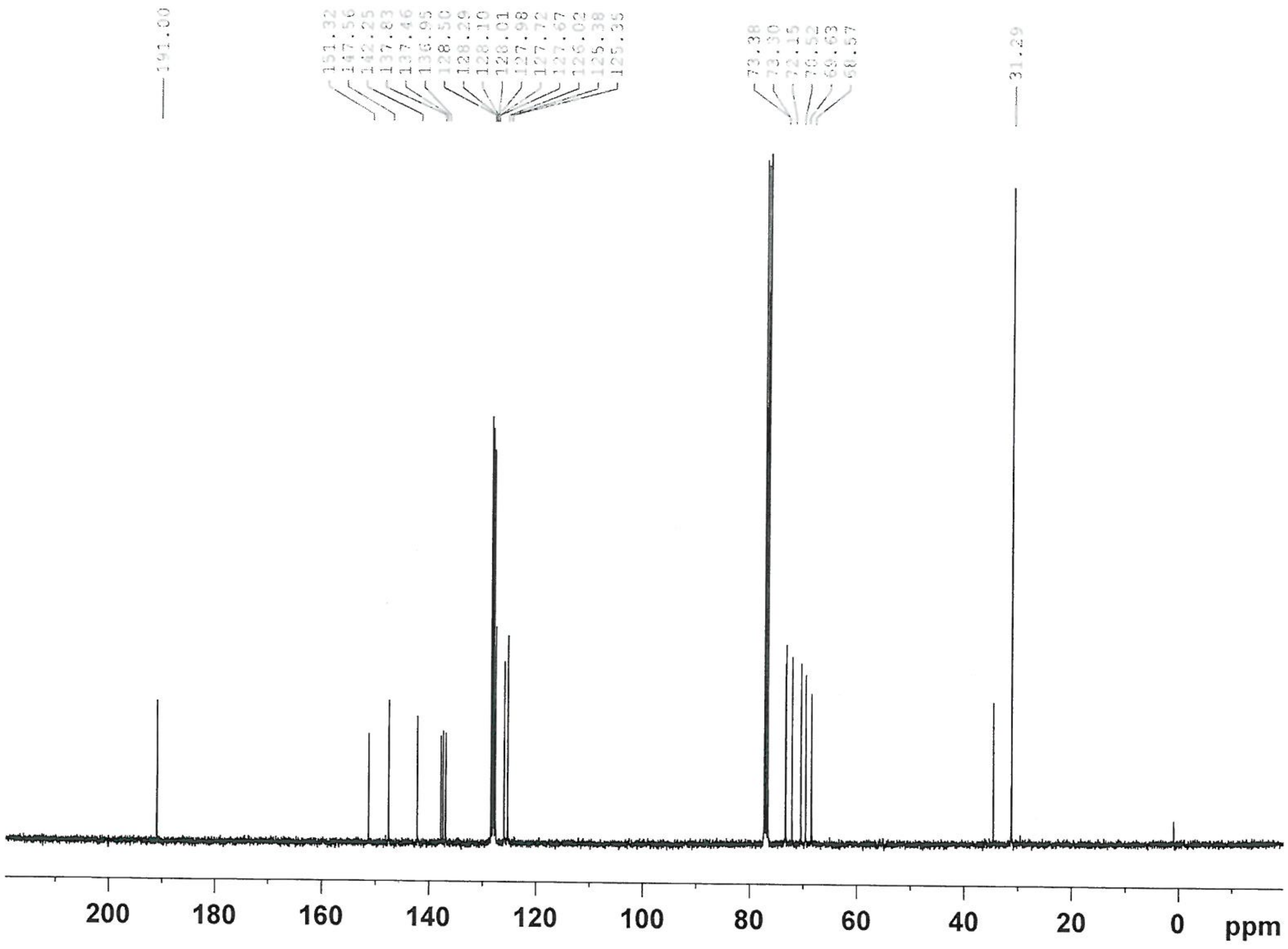

NAME	FERRIER_TRT_FINAL
EXPNO	11
PROCNO	1
Date_	20140625
Time	17.33
INSTRUM	spect
PROBHD	5 mm PABBO BB-
PULPROG	zgpg30
TD	65536
SOLVENT	CDCl3
NS	500
DS	4
SWH	24038.461 Hz
FIDRES	0.366798 Hz
AQ	1.3631988 sec
RG	101
DW	20.800 usec
DE	6.50 usec
TE	295.3 K
D1	2.00000000 sec
D11	0.03000000 sec
TD0	1

======== CHANNEL f1 ========

NUC1	13C
P1	10.00 usec
PL1	4.00 dB
SFO1	100.6328888 MHz

======== CHANNEL f2 ========

CPDPRG2	waltz16
NUC2	1H
PCPD2	90.00 usec
PL2	-3.00 dB
PL12	15.30 dB
PL13	18.00 dB
SFO2	400.1716007 MHz
SI	32768
SF	100.6[illegible]37 MHz
WDW	EM
[illegible]SB	0
LB	1.00 Hz
GB	0
PC	1.40

Central Analytical Facilities

CAF

STELLENBOSCH UNIVERSITY

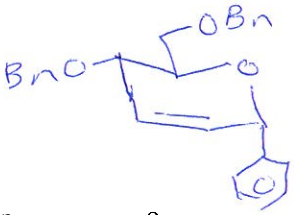

00000

9a

Requested: H. Kinfe (UJ)
Report No: HK_UJ_150211

Instrument: Waters Synapt G2
Introduction: ESI probe injected into a stream of acetonitrile with 5 % water.
Source: ESI positive, Cone Voltage 15 V
Lock mass: leucine enkephalin

Elemental Composition

File Edit View Process Help

Single Mass Analysis
Tolerance = 80.0 PPM / DBE: min = -1.5, max = 100.0
Element prediction: Off
Number of isotope peaks used for i-FIT = 3
Monoisotopic Mass, Even Electron Ions
219 formula(e) evaluated with 16 results within limits (all results (up to 1000) for each mass)
Elements Used:

Mass	Calc. Mass	mDa	PPM	DBE	Formula	i-FIT	i-FIT Norm	Fit Conf %	C	H	N	O
415.1902	415.1909	-0.7	-1.7	14.5	C27 H27 O4	248.5	1.653	19.15	27	27		4
	415.1882	2.0	4.8	15.5	C23 H23 N6 O2	250.2	3.377	3.41	23	23	6	2
	415.1923	-2.1	-5.1	19.5	C28 H23 N4	249.5	2.614	7.33	28	23	4	
	415.1981	-7.9	-19.0	10.5	C21 H27 N4 O5	250.9	4.072	1.70	21	27	4	5
	415.1810	9.2	22.2	19.5	C29 H23 N2 O	249.2	2.402	9.05	29	23	2	1
	415.2022	-12.0	-28.9	14.5	C26 H27 N2 O3	249.1	2.257	10.46	26	27	2	3
	415.1770	13.2	31.8	15.5	C24 H23 N4 O3	249.9	3.038	4.79	24	23	4	3
	415.2062	-16.0	-38.5	18.5	C31 H27 O	249.7	2.859	5.73	31	27		1
	415.1730	17.2	41.4	11.5	C19 H23 N6 O5	251.6	4.756	0.86	19	23	6	5

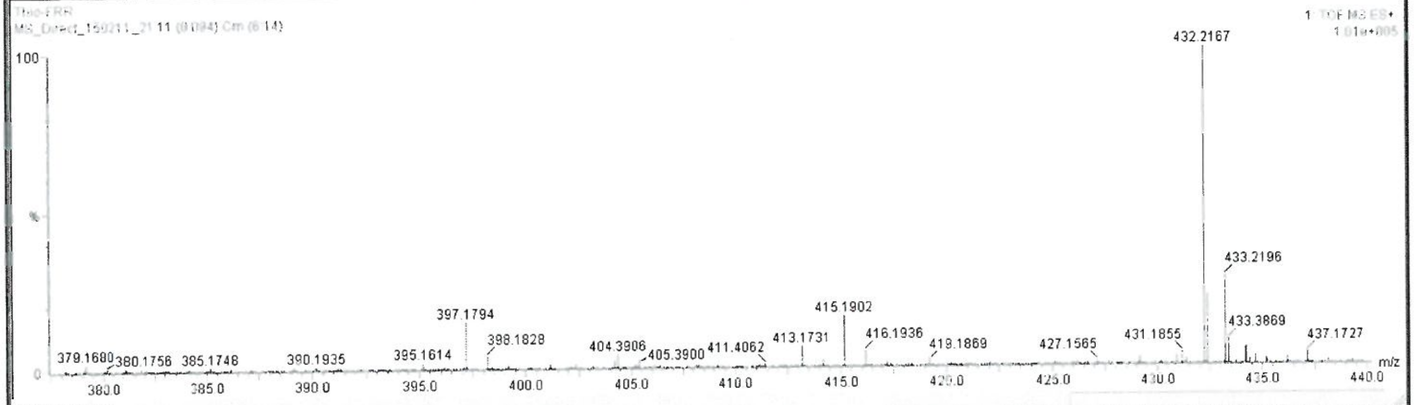

For Help, press F1

MS Unit

JC Smuts building., Private Bag X1, Matieland, 7602, South Africa

Tel: +27 21 808 5825 Fax: +27 21 808 5863 lcms@sun.ac.za www.sun.ac.za/caf
